# Batch correction methods for nontarget chemical analysis data: application to a municipal wastewater collection system

**DOI:** 10.1007/s00216-023-04511-2

**Published:** 2023-01-11

**Authors:** Madison E. Hattaway, Gabrielle P. Black, Thomas M. Young

**Affiliations:** Department of Civil and Environmental Engineering, University of California, Davis, Davis, CA 95616 USA

**Keywords:** Wastewater treatment, Mass spectrometry, Mathematical methods, Monitoring, Ions

## Abstract

Nontarget chemical analysis using high-resolution mass spectrometry has increasingly been used to discern spatial patterns and temporal trends in anthropogenic chemical abundance in natural and engineered systems. A critical experimental design consideration in such applications, especially those monitoring complex matrices over long time periods, is a choice between analyzing samples in multiple batches as they are collected, or in one batch after all samples have been processed. While datasets acquired in multiple analytical batches can include the effects of instrumental variability over time, datasets acquired in a single batch risk compound degradation during sample storage. To assess the influence of batch effects on the analysis and interpretation of nontarget data, this study examined a set of 56 samples collected from a municipal wastewater system over 7 months. Each month’s samples included 6 from sites within the collection system, one combined influent, and one treated effluent sample. Samples were analyzed using liquid chromatography high-resolution mass spectrometry in positive electrospray ionization mode in multiple batches as the samples were collected and in a single batch at the conclusion of the study. Data were aligned and normalized using internal standard scaling and ComBat, an empirical Bayes method developed for estimating and removing batch effects in microarrays. As judged by multiple lines of evidence, including comparing principal variance component analysis between single and multi-batch datasets and through patterns in principal components and hierarchical clustering analyses, ComBat appeared to significantly reduce the influence of batch effects. For this reason, we recommend the use of more, small batches with an appropriate batch correction step rather than acquisition in one large batch.

**Graphical abstract:**

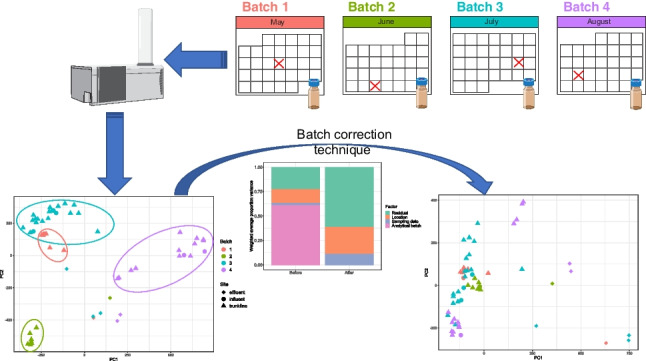

**Supplementary Information:**

The online version contains supplementary material available at 10.1007/s00216-023-04511-2.

## Introduction

High-resolution mass spectrometry (HRMS) has been applied to an increasingly diverse set of environmental problems extending far beyond its well-known ability to establish the presence of previously unmonitored compounds via suspect screening against mass spectral databases. Even without database matches or confident structural annotations, ions of particular mass-to-charge ratio (m/z) and chromatographic retention time (RT) observed across numerous samples can provide critical information about environmental processes, and such approaches fall within the domain of nontarget chemical analysis. A number of such applications rely on the comparison of ion abundances of unidentified compounds between samples collected at different times, monitoring locations or points in a treatment process [1–5]. Most of these studies have used patterns in feature abundance across samples to group and prioritize features for further identification efforts (e.g., features that decrease or increase across a treatment process or are present at higher abundance in particular types of sources). When the inverse question is posed—how samples may be grouped according to abundance profiles of multiple features (i.e., a chemical fingerprint)—the temporal or spatial differences in chemical composition under investigation may be masked by “obscuring variability” [6], or unintentional variability added during analysis.

This variability can come from a variety of sources that cannot always be controlled, such as operator effects, variations in the ion source, recent instrument maintenance, and sample-specific matrix effects [6, 7]. Effects of these variables can become the major driving factor in separation of sample groups in a method such as principal component analysis (PCA), rendering it less informative about chemical similarities and differences among samples [8]. Furthermore, in environmental monitoring applications that span periods of months to years, there will be batch effects regardless of whether samples are run in a single batch at the end of the study (due to differential degradation of compounds within extracts stored for varying periods) or in multiple batches as they are collected and processed (due mostly to instrumental variation). The ability to disentangle potential “batch effects” from the true chemical differences between samples is critical to drawing proper conclusions from the data.

While these challenges have already been addressed extensively in the fields of DNA microarrays, metabolomics, lipidomics, and others, environmental samples pose a unique set of challenges, delineated by Boysen et al. [9]. For example, complex and variable matrices can affect the ionization efficiency of compounds in inconsistent ways. In nontarget analysis of environmental samples, while there may be a group of constituents that are consistently detected, “contaminants of concern” may only appear sporadically or only at a specific site. This differs from a DNA microarray study, which employs a predetermined number of probes. The large proportion of sparse features in an environmental dataset can also make statistical analysis challenging. Environmental contaminants span a vast range of structural classes and are subject to significant fluctuation and alteration given changing consumption patterns and the advent of new compounds. Many environmental monitoring studies, mostly focusing on wastewater-impacted surface waters, have employed clustering methods on nontarget features to separate features into groups defined by spatial, temporal, or usage trends [2, 10–12]. Alternatively, studies aiming to identify new or site-specific contaminants focus on features with high intensity and low detection frequency [4, 13]. Of these six particular studies, only two reported employing intensity normalization to isotopically labelled internal standards. Furthermore, an in-depth review of study reporting in eight nontarget papers found that despite the sensitivity of nontarget analysis to analytical sequence, this aspect of data acquisition was “not adequately emphasized” [14].

Methods of correcting for batch effects have been categorized as quality-control (QC)-based, isotopically labelled, internal standard-based (ISTD), or sample data-driven [15]. QC-based methods rely on QC samples created from aliquots of all samples within a batch, which are injected multiple times throughout the course of a run. Then, models are used to find relationships between the QC peak intensities and injection order to separate batch effects from the biological/chemical differences [16, 17]. ISTD-based approaches employ a robust suite of isotopically labelled internal standards added to each sample, and feature peak intensities are scaled according to the corresponding ISTD. The method developed by Boysen et al. (2018) is a combination of both QC-based and ISTD-based methods, using replicate injections of pooled QC samples to determine the ISTD to best minimize the coefficient of variation for each feature. Compared to QC- or ISTD-based approaches, sample data–driven approaches have the benefit of avoiding the extra cost of internal standards and the need for additional instrument time [15]. One example of a sample data–driven method is ComBat, an empirical Bayes method that estimates the hyperparameters for the distribution of batch effects by pooling information across features within a batch, and then adjusts intensities accordingly [18]. Selection of the method of correction should be made based on the type of data collected and the nature of subsequent analyses to be performed. PCA, which performs a change of basis using variables explaining the greatest variation within the dataset, may become scrambled, whereas the results of a hierarchical cluster analysis, which considers the similarities between samples or features, may become clearer [19].

Because batch correction can introduce bias and variance, it is also necessary to identify measures of the “success” of batch correction approaches. These measures are borrowed from the ‘omics fields that have addressed this issue previously, although they require special considerations when applied to environmental samples. For example, a visualization technique such as relative log abundance (RLA) plots, which centers features either by the within- or across-group median and uses boxplots to assess the “tightness” of features around zero must presume that metabolites will be present in every sample [20]. This method would not necessarily be applicable to sparse environmental datasets, especially ones geared towards the discovery of contaminants of concern unique to a site or sampling date. If we were to assume that (high-quality) features that are detected in more than 85–90% of samples are part of a consistent background metabolome, then those features may be visualized via RLA plots for comparison between correction methods. Both De Livera et al. [20] and Drotleff and Lammerhofer [21] recommend use of multiple methods to assess batch correction efficacy, combined with a holistic evaluation rather than quantitative thresholds. A method known as principal variability component analysis (PVCA) estimates the variability within the dataset that is associated with analytical batches by fitting a linear mixed model to the first few principal components [22, 23]. With this method, seeing a decrease in the variability associated with analytical batch from the uncorrected to the corrected data would suggest a successful correction.

Given the challenge posed by batch effects for nontarget studies that monitor complex environmental matrices over extended periods of time, this study aims to assess the applicability of batch correction techniques and the measures of their success. While there is not a one-size-fits-all approach for different datasets, this can still serve as a relevant example of the process. To this end, we used multiple datasets: (1) wastewater treatment plant (WWTP) influent and effluent samples plus sites within the catchment system collected over a 9-month period and analyzed in four different analytical batches as the samples were processed (multi-batch; MB), and (2) the same wastewater samples run in a single analytical batch (single batch; SB), after all the samples had been collected and processed (Table 1). ComBat correction, an empirical Bayes technique, was applied to the MB dataset and the results were compared to the uncorrected MB and SB datasets using principal variation component analysis (PVCA), principal component analysis (PCA), and hierarchical clustering analysis (HCA). Additionally, a conventional approach of using sample median internal standard (ISTD) peak heights to scale raw peak heights of features was employed on the same MB dataset to obtain MB-IS.

**Table 1:** Description of datasets and processing applied to each

Sample description	Dataset name		Analytical batches	Analytical run date	Samples per analytical batch	Processes applied
- Sub-sewershed (*n* = 42) - WWTP influent (*n* = 7) - WWTP effluent (*n* = 7)	SB		1	7/17/17	56	- Feature filtering - Log2 transform - Quantile normalization
	MB-unC	4		5/22/16	8	“”
				6/17/16	8	
				9/26/16	24	
				3/3/17	16	
	MB-IS	“”		“”	“”	- Feature filtering - Scaled by median ISTD peak height
	MB-C		“”	“”	“”	- Feature filtering - Log2 transform - Quantile normalization - ComBat correction

## Materials and methods

Detailed descriptions of the sample collection, preparation, data acquisition methods, and quality assurance/quality control measures applied to this sample set are reported elsewhere by Budd et al. (in review) [24] and are summarized in the associated supporting information file. The NTA Study Reporting Tool (SRT) was used in the preparation of this manuscript [14].

### Nontarget alignment

First, raw data files were converted from instrument vendor format (Agilent.d files) to the analysis base file format (Reifycs Analysis Base File Converter v. 4.0.0). These files were then deconvoluted and aligned in MS-DIAL (v. 3.90) using internal standards for retention time correction. The data files included in the MB alignment were as follows: 7 method blanks (one per month), 10 wastewater matrix spikes (one each month plus three extra), 4 100 ppb calibration standards (one per batch), 7 influent samples, 7 effluent samples, and 75 sub-sewershed samples. The data files included in the SB alignment were as follows: 8 method blanks, 12 wastewater matrix spikes, 7 100 ppb calibration standards, 7 influent samples, 7 effluent samples, and 74 sub-sewershed samples. For SB, conventional LC–MS parameters were used, whereas for MB-unC/C, an All-Ions experiment file was included. Alignment parameters are detailed in the supporting information, Tables S1, S2, and S3.

### Data pre-processing

To remove low abundance background features, filtering rules were applied. A standard signal-to-noise cut-off of ten was used. A blank filter required that the maximum signal in wastewater samples be greater than ten times the average signal in the method blanks. Features with retention times below 4.5 min were excluded due to the poor chromatographic quality of early-eluting peaks that results in unreliable alignment of these features.

To further reduce the number of features, Mass Spectral Feature List Optimizer (MS-FLO), which was designed as post-processing step to be used with MS-DIAL alignments [25], was implemented to join ammonium and sodium adducts to their matching molecular ion. Parameters used are included in Table S4. Next, features that were not detected at a height of at least 3000 counts in at least one of the 56 WW samples were eliminated, as some features were only present in blank, standard, or spiked wastewater samples that were included in the alignment set for quality control purposes. The phenomenon of split features, which can occur during alignment despite the use of retention time correction from labelled internal standards, was handled by joining features that met the following three criteria: (1) retention times within ± 15 s, (2) m/z within 10 ppm, and (3) MS1 isotopic abundance ratios with coefficient of variation of 20% or less. Finally, features that occurred in 60% or more of samples from at least 1 month or site were retained, as the study goal was to find patterns of chemical profiles in space and time. After all feature filtering steps, the sample set was reduced to only include 56 wastewater samples: 7 influent, 7 effluent, and 42 trunkline samples, to enable better comparison between SB, MB, and the work done by Budd et al. [24].

To obtain the dataset MB-IS, every peak height was divided by the median ISTD peak height of the sample, using the labelled ISTDs that were also used for retention time correction in the MS-DIAL alignment (Table S3). This was used rather than a retention-time specific ISTD correction, because of the uneven distribution of ISTDs throughout the duration of the chromatographic run.

Additionally, raw height values were transformed by applying ${y}^{^{\prime}}={\mathrm{log}}_{2}(y+1)$, since ion abundances for over 90% of features in this dataset range over more than six orders of magnitude. Then, for all datasets, quantile normalization was applied to adjust the distributions of feature heights [26]. Quantile normalization was implemented in R using *normalize.quantiles* from the package *preprocessCore* (v. 1.54.0) [27].

### Batch correction

Briefly, ComBat assumes that the additive and multiplicative batch effects on each feature are part of a distribution of batch effects. The estimated batch effects did not appear to fit within the assumed distributions used by the parametric ComBat method; therefore, the nonparametric method was used. The method can pool information across features to estimate the additive and multiplicative effects, and adjust intensities accordingly.

The nonparametric ComBat batch correction method [18] was applied to MB-unC to create MB-C. Covariates indicating experimental factors for site and sampling date were not applied. ComBat was applied in R using *ComBat*, part of the *sva* (v. 3.40.0) package [28].

### Data analysis (PCA, HCA, PVCA, and differential abundance)

The script for conducting PVCA was adapted from Boedigheimer [23]. Experimental factors included in the model were sampling location, sampling date, and analytical batch number (all variables as factors). The script first calculates the correlation matrix for the dataset, then uses *lme* (from package *lme4* v. 1.1.27.1) [29] to fit a linear mixed effects model using the experimental factors for each principal component, up to 60% of overall variance, where *pc*_*n*_ is the number of principal components required to account for 60% of overall variance. For the three multi-batch datasets, the model formula used was as follows:

1 $${pc}_i\sim\left(\left.1\right|Sampling\;month\right)+\left(\left.1\right|Sampling\;location\right)+\left(\left.1\right|Analytical\;batch\right)$$

where the mean of each sampling month, sampling location, and analytical batch was assumed to be randomly distributed with a center of zero and an unknown variance. For the single-batch dataset, the analytical batch term in Eq. 1 was omitted. The mixed effects model pools information within each factor to compute an unbiased estimation of the variance. The weighted average of variance for each experimental factor is then computed using eigenvalue of each *pc*_*i*_ as the weight. Matrices of the final 56 samples containing features that had been filtered, joined, quantile normalized, and (for MB-C only) ComBat corrected, were visualized using PCA. For hierarchical clustering, the same *pc*_*n*_ components used to account for 60% of the dataset variance used in PVCA were included, using Euclidean distance and the Ward’s agglomeration algorithm [2, 11]. Number of clusters was selected after consideration of silhouette [30] and gap-statistic [31] plots, commonly used to determine optimal number of clusters.

Differential abundance of features compared across sampling months and sites was analyzed with the *limma* (v. 3.48.3) package [32] in R, using a similar approach to differential expression analysis for DNA microarrays [32, 33]. This method uses the function *lmFit* to compute a model for each feature according to experimental design groups. Modelling interactions between month and site was not possible since a single sample was taken for each month-site combination. Contrasts were defined to determine fold change and significance between experimental groupings of month, site, and cluster (as defined by HCA). For example, the contrast “Influent – Effluent” compares the abundances of features between influent and effluent samples. To compute statistical significance, the *eBayes* function was used, which employs an empirical Bayes approach to shrink standard errors of features toward a pooled estimate [34]. The Benjamini–Hochberg correction was used to control the false discovery rate [35], such that features with an adjusted *p*-value less than 0.05 were retained for analysis.

## Results and discussion

### Feature filtering and joining

After applying filtering rules, the number of features decreased from 63,259 to 3108 in SB and 136,938 to 25,822 in MB (Table S6).

One possible reason for this discrepancy may be the degradation of some features during sample storage before the acquisition of the single-batch data. The extent of this degradation may depend on the types of compounds present in samples, as it has been found that illicit drugs, pharmaceuticals, and their metabolites are stable when stored on SPE cartridges at − 20 °C [36, 37], whereas some antibiotics degrade in extracts at − 20 °C after 4 weeks [38]. Another possible explanation is that the SB dataset contains only MS1 information, whereas the MB dataset was acquired in *All-Ions* mode. This could lead to MS-DIAL misidentifying fragments as molecular ions. Finally, the use of multiple analytical batches could simply lead to more misalignment of features due to greater mass error and contamination from samples run between batches.

In addition to adducts making it through the alignment algorithm, split features are also often observed. These can take one of two forms: duplicate features and alternating features. Duplicate features have identical abundance values for most of the samples, but the algorithm missed the abundances for one of the features in a handful of samples so these are reported as two features (Table 2a). MS-FLO incorporates a duplicate feature joining tool, but duplicate features are not always consolidated by this application. The second form of split features, alternating features, results in abundances of the same magnitude being reported for every other feature across samples (Table 2b). These split features are hypothesized to be artifacts from the retention time correction step in alignment, which should affect single-batch date less than multiple-batch data. The reduction in feature number after the process of joining split features was 6.7% (2126 features) for SB and 5.5% (3847 features) for MB.

**Table 2:** Example application of split feature joining algorithm

					Abundance							
	Alignment ID	Average m/z	Average RT	M:M + 1 (or M + 2)	Sample 1	Sample 2	Sample 3	Sample 4	Sample 5	Sample 6	Sample 7	Split feature type
a	141	83.0501	5.82	0.060	165,052	119,320	114,649	66,851	149,155	108,584	56,291	Duplicate feature
	142	83.0502	5.7	0.058	165,052	119,320	114,649	66,851	149,155	108,584	56,291	
b	185	89.06	6.07	0.046	903,124	0	723,861	77,930	0	0	69,978	Alternating feature
	187	89.0601	5.85	0.050	560	541,018	723,861	568	641,555	437,522	530	

Ultimately, joined split features made up 5.1% (about 1316 features) of MB and 24% (about 746 features) of SB. The existence of split features or similar alignment artifacts is a challenge that is not unique to our lab group or users of MS-DIAL. For example, Schollée et al. [1] employed a similar algorithm of joining features when they faced the issue of peak tailing being identified as unique features when using *enviPick* for alignment.

### Comparison of principal variance component analysis

**Fig. 1:**
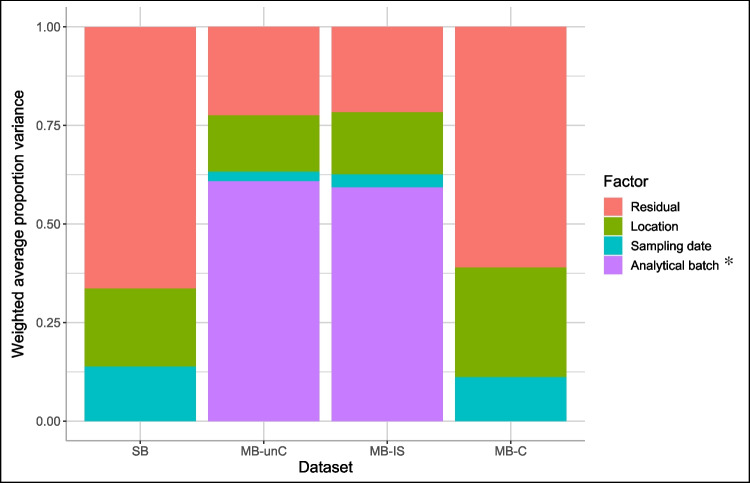
PVCA plots show contributions to the overall variance for three experimental factors plus unexplained (residual) variance for single-batch (SB), uncorrected multi-batch (MB-unC), ISTD-scaled multi-batch (MB-IS), and ComBat corrected multi-batch (MB-C). The asterisk symbol indicates the analytical batch factor was omitted for SB

PVCA allows for visualization of the contributions of experimental factors (sampling location, sampling date, and analytical batch) as well as the proportion of residual, or unexplained variance, to overall variance within the dataset (Fig. 1). For SB, we included a simulated analytical batch, which assigned SB samples to the analytical batch number in which it was analyzed in the MB datasets. The weighted average proportion variance of 0.15 computed for batch is slightly higher than for sampling date, but lower than for sampling location or residual. With this sample set, it is difficult to separate the factors of sampling date and analytical batch, because samples from the same month are always in the same batch, and this collinearity is likely the reason for the variability attributed to simulated analytical batch here. For MB-unC, analytical batch is associated with the greatest proportion of the variance, which then decreases after ISTD scaling is applied (MB-IS) and goes away completely with the application of ComBat correction (MB-C). Instead, for MB-C, much of the variability in the dataset is “residual”, meaning it cannot be attributed to sample date or location alone.

### Comparison of principal component analysis and hierarchical clustering analysis

The change seen in the PVCA plots is consistent with the way that samples rearrange after PCA and HCA. For set SB, the plot of the first two principal components (Fig. S3a) shows effective separation of effluent samples from influent and trunkline samples in PC1. Further inspection via HCA (*pc*_*n*_ = 16) reveals separation of six effluent samples plus July influent from the rest of the sample set (Fig. S3b) in clusters 1 and 2, while the July effluent sample is found in cluster 4. Mixing of months is observed in all clusters. There is some grouping by sampling site, for example in cluster 3, there are five samples from site E and four from site G grouped together. Examination of principal component pair plots of PCs 1 through 5 (Fig. S5a) shows a consistent mix of samples from different sampling dates and analytical batches (where a simulated analytical batch variable is included for the sake of comparison).

**Fig. 2:**
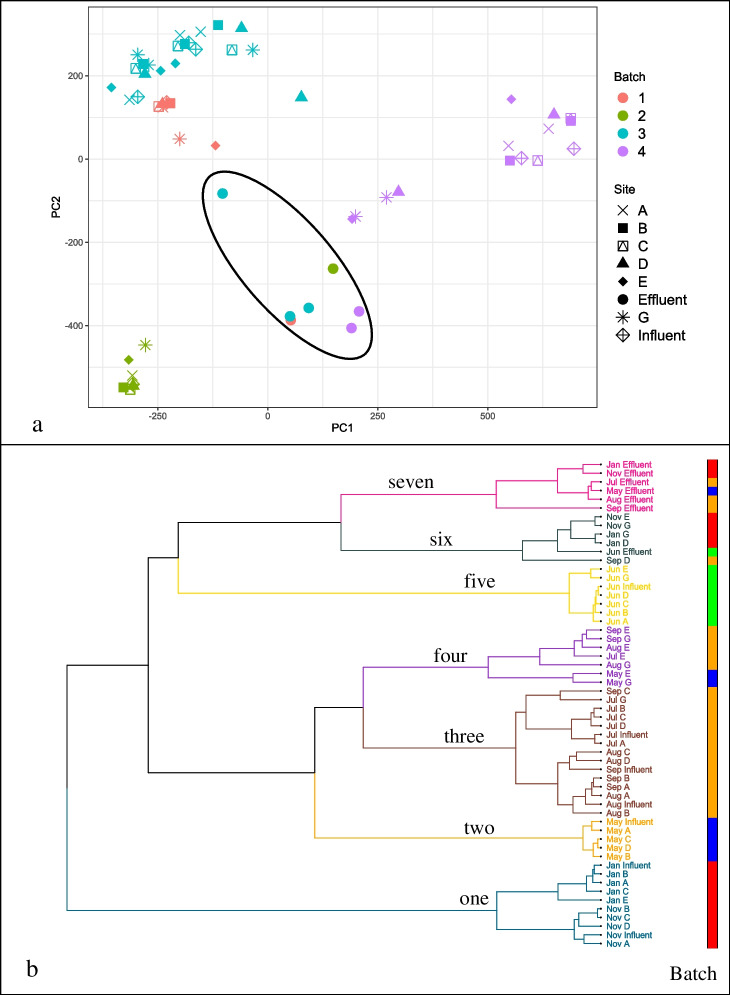
For MB-unC, (**a**) plotting of first two principal components revealed clustering was driven by analytical batch, with effluent samples delineated by black oval, and (**b**) HCA (*pc*_*n*_ = 4), divided into seven clusters, numbered one through seven for ease of discussion. The colored bar to the right of the dendrogram is colored according to analytical batch of each sample: blue for batch 1, green for 2, orange for 3, and red for 4. Cluster 1 is entirely batch 4 samples, two 100% batch 1, three is 100% batch 3, four a mix of 1 and 3, five 100% batch 2, six is combination of batches, and seven is all effluents except June (batch 2)

For the MB-unC PCA plot, apart from a cluster of effluent samples, there is clear evidence of separation of samples by batch (Fig. 2a): samples from batches 1 and 3 group in the top-left corner, batch 2 in the bottom-left, batch 4 in the top-right, and effluent samples in the lower middle. Although they do form a relatively distinct cluster, the effluent samples are not clearly distinguished from influent/trunkline samples along either the first or second principal component axes, which account for the greatest percentage of overall variance. This indicates that before batch correction, analytical batch differentiates samples more than whether they were treated or untreated wastewater. November and January, the fourth analytical batch, group together, making up the entirety of cluster 1 in the HCA (Fig. 2b). June, the second analytical batch, is also on its own, comprising the entirety of cluster 5 in Fig. 2b. Interestingly, the third batch, consisting of months July, August, and September, groups close with the first batch (May), forming clusters 2, 3, and 4. While cluster 2 is comprised entirely of May samples, two May lateral sites, E and G, mix with E and G samples from August, July, and September in cluster 4. Higher-order principal components show the separation of May samples from other months, in Fig. S3b. The only variation from the batch-wise segregation of samples can be seen in the clustering of September D, November G/E, and January D/G trunkline samples with June effluent (Fig. 2b, cluster 6), and a group of May, July, August, and September samples of only sites E and G (Fig. 2b, cluster 4).

In the MB-IS dataset, feature peak-heights were scaled by the median internal standard peak height in each sample. The resulting plot of the first two principal components (Fig. S4a) shows that the influent and trunkline samples are grouped again by batch, with batch 3 on the left, batch 1 in the middle, batch 4 in the upper right corner, and batch 1 in the lower right corner. On the first principal component axis, the treated effluent samples are in line with the untreated samples from batches 1 and 4. This is also seen in Fig. S4b, where only 2 clusters, 6 and 7, contain samples from different batches. For this dataset, the conventional remedy for batch effects, scaling by internal standards, is not enough.

**Fig. 3:**
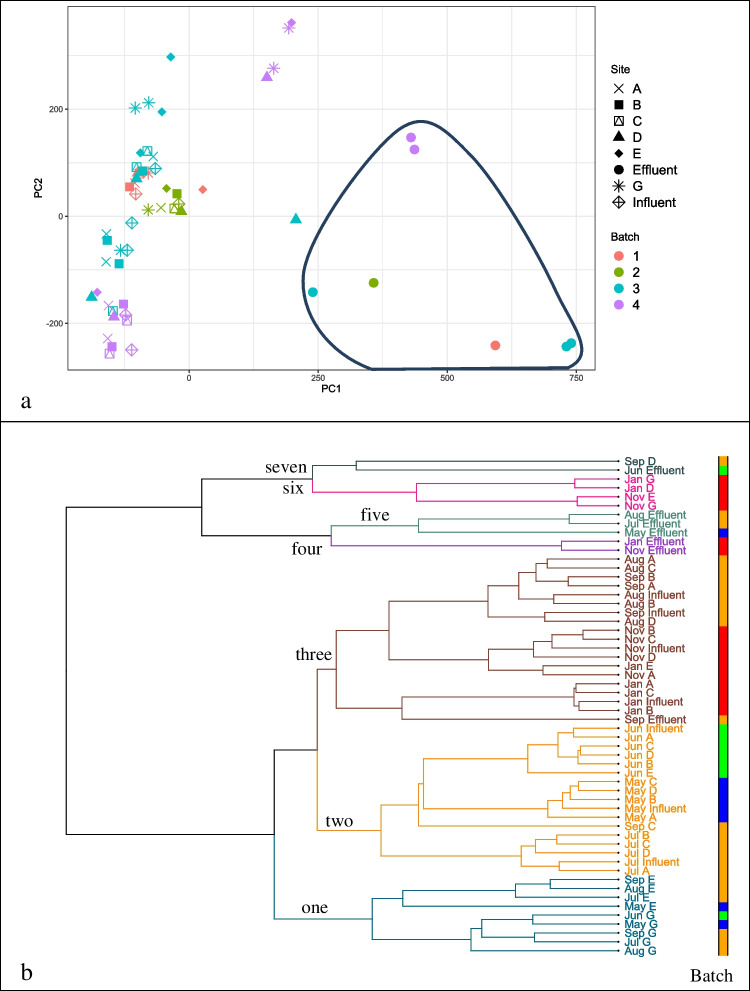
For MB-C (ComBat corrected MB-unC), (**a**) plotting the first three PCs shows absence of batch-wise separation observed in Fig. 2a (again with effluent samples circled) and (**b**) HCA (*pc*_*n*_ = 13) shows more mixing of samples of different batches in the same cluster: cluster 2 contains samples from batches 1, 2, and 3. Analytical batch is indicated by colored bar to the right of the dendrogram as in Fig. 2b

After applying the ComBat batch effect correction method to MB-unC, the grouping of samples through PCA and HCA changed (Fig. 3a, b). In the graph of the first three principal components, now most samples group together, although the group of effluent samples is clearly separated primarily by PC1. Additionally, the clustering of analytical batches that was observed previously in HCA is less apparent: May (batch 1) and June (batch 2) samples are now contained within cluster 2 along with July (batch 3) samples. November and January (batch 4) samples, which previously had their own cluster, are now grouped into cluster 3, along with September and August (batch 3) samples. The same September D, November G/E, and January D/G trunkline samples that made up cluster 6 in Fig. 2b are still grouped close to the effluent samples (clusters 6 and 7, Fig. 3b). Cluster 1 now only contains the E trunkline samples that were previously with G trunkline samples in cluster 4 for MB-unC. Additional principal components bear out this increased mixing of samples between months, in Fig. S4d.

As a final point of comparison, the same sample set was clustered according to concentrations of target pesticides as reported by Budd et al. (in review) [24]. While the dataset of quantified target pesticides considers a significantly smaller subset of compounds (nineteen pesticides) than the nontarget features, this data has been matrix corrected, which is difficult to achieve with nontarget data. Only two of the target compounds can be measured via LC–MS (fipronil and imidacloprid), while the rest were measured via GC–MS. However, we hypothesized that the adjuvants used in commercial formulations could create an LC-detectable source signature of the pesticide.

From this analysis, August G, November A and E, and January B and D all were differentiated from the bulk of the samples (Fig. S6d). Clearly, the August G sample is unique, because it is the only sample with a quantifiable amount of chlorothalonil. November A is the only sample in the set with quantifiable esfenvalerate, while November E is unique for the significantly higher concentrations of the pyrethroids, cypermethrin, deltamethrin, and permethrin, which were frequently detected across sites and months. January D contained a higher concentration of cyfluthrin, also frequently detected across sites and months, and January B and D both had much higher concentrations of fipronil amide.

Similarly, in the nontarget MB-unC/C analyses, November E and January D were distinguished in HCA, which would suggest that the nontarget approach is also able to pick out unique samples despite the higher background noise of nontarget features. Recall that for both MB-unC/C, November G, and January G samples were included in a distinct cluster with November E and January D, perhaps indicating an additional chemical similarity between these samples. For SB, there was some correspondence with the target data with some separation of “distinctive” samples, with the distinctive groups made up of five E (including November) and four G samples (including August, November, and January). A different grouping of four E samples was observed in both the MB-unC (cluster 4) and MB-C (cluster 2) datasets. The site E catchment area has a much higher percentage of high-density residential zoning than other sites, which Budd et al. (in review) [24] connected with this site’s significantly higher loadings of pyrethroids. Presumably, the higher density of people could lead to a distinct nontarget signature from associated with higher loadings of pharmaceutical and personal care products as well.

However, other samples that were unique in their target pesticide concentrations were not found to be as unique when considering nontarget features. January B was grouped near January influent, A, and C in HCA for SB, MB-unC, and MB-C, perhaps indicating that the bulk of the nontarget features for this sample outweighed whatever differences result from the high concentrations of target pesticides. This may be true for November A and August G as well, which were distinguished from the target data as the single quantifiable detections of esfenvalerate and chlorothalonil, respectively, do not show consistent separation in the nontarget datasets.

### Comparison of differential abundance

Evaluation of features found to be significantly different between sampling dates or sampling sites was carried out for MB-unC and MB-C. There were a total of 21 contrasts for comparing abundance by month, and 28 by site. Additionally, 21 contrasts were created by dividing samples according to clusters determined using HCA (Figs. 2b and 3b). Using the within-cluster sum of squares and gap-statistic methods, the optimal number of clusters was between 7 and 10, so the number of 7 clusters was selected (Figs. S9–S12). It is important to note that the “one-two” contrast for MB-unC does not consist of the same samples as the “one-two” contrast for MB-C (and so on) because of different HCA results.

In set MB-unC, features with an adjusted *p*-value < 0.05 were found for all 21 contrasts comparing months. Only 5 comparisons of months had significantly different features for MB-C: August–July, September–July, January–November, September–August, and January–July. Table S7 summarizes the number of significantly different features found for each dataset and contrast. As illustrated in Figs. S13 and S16, *p*-value distribution shapes differed considerably between MB-unC and MB-C. The sharp drop-off from the left to right in many of the contrasts in the MB-unC set indicates a higher number of significantly different features. A small subset of features was found to be significant before and after ComBat correction: 8 features for January–November, 4 for September–August, 126 for August–July, 3 for January–July, and 80 for September–July.

While 23 out of the 28 site-wise contrasts returned significantly different features for MB-unC, no significantly different features were found using site-wise contrasts for MB-C. While some studies have found that a drawback of applying ComBat is the generation of false positive data [39, 40], we found that applying ComBat in this study may have actually lessened differences between samples. Indeed, others have found that applying ComBat with an unbalanced experimental design can deflate significance [41], but in an environmental monitoring application, the relationship between sampling date and run date is intractable. Furthermore, the assumption of the algorithm itself that “phenomena resulting in batch effects often affect many genes in similar ways (i.e., increased expression, higher variability, etc.),” [18] may be appropriate for DNA microarrays, but not for the behavior of compounds with unknown physicochemical properties analyzed by the LC-QTOF-MS. It is also possible that many of the features that collectively constitute the “wastewater metabolome” simply do not vary much between months and sampling sites.

Comparison of the HCA clusters for the ComBat corrected dataset was slightly more successful than comparison by month or site. Indeed, in the analysis of target pesticides, it is evidenced that there can be considerable variation within sampling dates and sampling locations. For example, there were many significantly different features within the effluent samples, contained in clusters 4, 5, and 7. Creating an m/z vs RT plot of features significant to each cluster (Figs. S21 and S22) revealed a large swath of features that could be composed of homologous series that were significant to cluster 7 (June effluent and September D). No features were found to be significant to the largest cluster, 3, perhaps because there was still too much variability between the samples (19 out of the 56 samples). As was the case with comparison by site and by month, many more features were found to be significant for MB-unC clusters. The swath of (possible) homologous series in the effluent samples was present in this dataset as well (Figs. S19 and S20).

### Recommendations

We have shown with these datasets that batch effects from multiple analytical runs can be examined through PVCA, PCA, and HCA, and the novel application of ComBat can reduce the obscuring effect on the overall spatial and temporal differences in the data. Given the choice between analyzing samples in multiple analytical batches or a single analytical batch over the course of a long-term environmental monitoring study, we would recommend the use of multiple batches with the application of a method such as the one demonstrated here. Further recommendations would include additional QA/QC measures that we did not have at the time of data acquisition for this study, such as replicate injections of pooled matrix spikes for each class of matrix (for example, the trunkline/influent would be a separate matrix spike from the effluent). Furthermore, a more robust standard mix of labelled internal standards with improved coverage of the range of retention times and physicochemical properties could enable ISTD-based batch correction approaches, which may be more appropriate for MS data.

## Supplementary Information

Below is the link to the electronic supplementary material.

Supplementary file1 (PDF 2613 KB)

Supplementary file2 (XLSX 50 KB)

## Data Availability

R files used in data processing available at https://github.com/madaway/WW_Batch_Correction. Original multi-batch alignment available at https://doi.org/10.5281/zenodo.7097871; original single-batch alignment available at https://doi.org/10.5281/zenodo.7097911.
