# Supplementary material for: Batch correction methods for nontarget chemical analysis data: application to a municipal wastewater collection system
Source: Anal Bioanal Chem. 2023 Jan 11;415(7):1321–31. doi: 10.1007/s00216-023-04511-2 (PMC9928919; doi:10.1007/s00216-023-04511-2)
Supplement: Supplementary file 1 — Supplementary file1 (PDF 2613 KB) [file 216_2023_4511_MOESM1_ESM.pdf]

Supporting Information

Batch Correction Methods for Nontarget Chemical Analysis Data: Application to a Municipal Wastewater Collection System

Madison E. Hattaway, Gabrielle P. Black, Thomas M. Young

Department of Civil and Environmental Engineering, University of California, Davis

CONTENTS

List of Figures

Fig. S1 Schematic of connections within sewer system. 2
Fig. S2 PCA scree plots 9
Fig. S3 PCA, HCA for SB. 9
Fig. S4 PCA, HCA for MB-IS. 10
Fig. S5 Matrix pair plots 13
Fig. S6 Target pesticide PCA 14
Fig. S7 Target pesticide HCA. 15
Fig. S8 Top-ten highest variable contributions to pesticide PCA. 16
Fig. S9 Gap-statistic plot for MB-unC. 17
Fig. S10 Total within cluster sum of squares for MB-unC 17
Fig. S11 Gap-statistic plot for MB-C. 17
Fig. S12 Gap-statistic plot for MB-C. 18
Fig. S13. Histograms of p-values from contrasts by months of MB-unC 18
Fig. S14. Histograms of p-values from contrasts by sampling site of MB-unC. 19
Fig. S15. Histograms of p-values from contrasts by HCA cluster for MB-unC. 19
Fig. S16. Histograms of p-values from contrasts by month of MB-C 20
Fig. S17. Histograms of p-values from contrasts by site for MB-C. 20
Fig. S18. Histograms of p-values from contrasts by HCA cluster for MB-C. 21
Fig. S19 M/z to RT plot of features found to be significantly lower in a single cluster in MB-unC dataset. 23
Fig. S20 M/z to RT plot of features found to be significantly higher in a single cluster in MB-unC dataset. 23
Fig. S21 M/z to RT plot of features found to be significantly higher in a single cluster in MB-C dataset. 24
Fig. S22 M/z to RT plot of features found to be significantly lower in a single cluster in MB-C dataset.. 24

List of Tables

Table S1: LC-QTOF-MS method parameters 3
Table S2: MS-DIAL alignment parameters for SB and MB datasets 4
Table S3: Labelled internal standards used for retention time alignment in MS-DIAL 6
Table S4: Parameters used for MS-FLO for adduct feature joining 6

Table S5: Target compound detection in standards and spiked wastewater after MS-DIAL alignment 7
Table S6: Results of feature filtering rules 7
Table S2. Counts of features with $p_{adj} < 0.05$21 *23*

Supplementary Experimental:

Sample Collection

Briefly, samples were collected as twenty-four-hour time-weighted composites at the wastewater treatment plant influent, effluent, and six sub-catchment locations before the treatment plant.

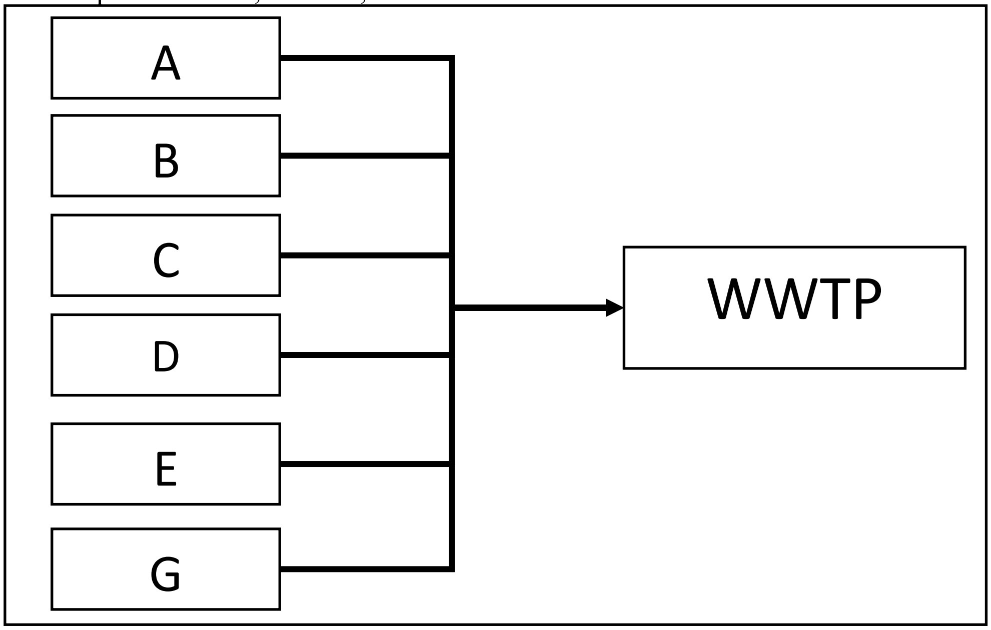

Fig. S1 Schematic of connections within sewer system. Trunkline sites A, B, C, D, E, G, plus combined influent and treated effluent of the WWTP were sampled monthly

Sample Preparation

Within 24 hours of collection, samples (200 mL of raw wastewater or 1 L of treated effluent) were filtered through a GF/F filter (0.45µm) which was extracted separately. Filtered samples were spiked with of a stable isotope labeled surrogate solution (Table S2), and passed over an Oasis HLB cartridge (Waters, Massachusetts, USA), then eluted ethyl acetate followed by methanol, which were collected separately. Jars were rinsed with 3:1 hexane: acetone, which was combined with the ethyl acetate eluate and concentrated to 1 mL. Methanol eluate was also concentrated to 1 mL. Filters were air-dried and then extracted in a sonicating bath with 1:1 hexane/acetone, then concentrated to 1 mL. The resulting extract for each sample was created from 0.5 mL of the ethyl acetate, methanol, and hexane-acetone extracts, then concentrated to 0.2 mL and reconstituted to 1 mL with ultrapure water and spiked with the stable isotope labeled internal standard mix.

Liquid Chromatography and Mass Spectrometry

Data was acquired using an Agilent 1260 Infinity HPLC coupled to an Agilent 6530 QTOF-MS. Chromatographic separation was achieved with a Zorbax Eclipse Plus C18 column (100 mm, 2.5 mm, 1.8 µm, Agilent Technologies, Inc.) Ultra-pure water plus 0.1% (v/v) formic acid (A), and acetonitrile plus

0.1% (v/v) formic acid (B) were used as mobile phases for positive electrospray ionization. The initial gradient was held at 2% B for 1.5 minutes, followed by a linear increase to 100% B at 16.5 min and held for 4 min. A postrun column equilibrium time of 3 minutes was used resulting in a total run time of 23.5 minutes. For the single batch set (SB), only MS1 data was collected. For the multi-batch set (MB-unC/C), acquisition was done using *All-Ions* fragmentation method. Extract sequence was not randomized, and every 10 to 15 extracts were followed by an injection of a solvent blank and replicate of a calibration standard. QTOF-MS operational parameters are included in Table S1 and were as described in Moschet et al. (1).

The same extracts were used to acquire MB and SB; vials were returned to the -20° C freezer within 24 hours of injection.

Method Evaluation, Quality Assurance, and Quality Control Measures

Prior to applying this analytical method to the wastewater samples tested here, the performance of the method was evaluated for 22 pesticides in wastewater influent samples analyzed in positive electrospray ionization mode. Recoveries of spiked compounds ranged from 57-120% with an average of 89% recovery, and method detection limits were between 4 and 34 ng/L. Quality assurance and quality control measures applied during the study included preparation and analysis of one method blank sample and one matrix spike duplicate in each monthly batch of 8 samples. Performance of the overall method for nontarget feature extraction was evaluated by assessing the ability to align, recover spectra, and identify compounds in the matrix spike samples. When spiked at 100 ppb into wastewater influent, the alignment and deconvolution algorithms correctly isolated and identified 100% of the spiked compounds in both single and multi-batch data sets for 19/23 compounds, with high detection frequencies for most of the remaining compounds and was over 50% effective for 18/23 compounds spiked at 20 ppb in both data sets (Table S4).

Table S1: LC-QTOF-MS Method Parameters

**LC Settings**		
Injection Volume		40 µL
Mobile Phases	A	miliQ water + 0.1% formic acid
	B	acetonitrile + 0.1% formic acid
Solvent Flow		0.35 mL/min
Gradient		2% B for 1.5 min
		2%-100% B in 15 min
		100% B for 5 min
		equilibration to initial conditions for 3 min
Column		Zorbax Eclipse Plus (100 mm length, 2.5 mm ID, 1.8 µm particle size)
Column Temperature		30°C
**MS Settings**		
Gas Temperature		300 °C
Dyring Gas Flow		12 l/min
Nebulizer		25 psig
Sheath Gas Temperature		350 °C
Sheath Gas Flow		11 l/min
Vcap		3500 (pos), 3000 (neg)
Fragmentor		110 V
scan range		50-1050 m/z
scan speed		4 spectra/s

All-Ions Acquisition	Collision Energy (CE): 0, 10, 20, 40
Reference Mass Correction	pos: 121.0509, 922.0098

Table S1: MS-DIAL alignment parameters for SB and MB datasets

	**SB**	**MB-C; MB-UnC**
**Project**		
MS1 Data type	Profile	Profile
MS2 Data type	Centroid	Profile
Ion mode	Positive	Positive
Target	Metabolomics	Metabolomics
Mode	ddMSMS	diMSMS
**Data collection parameters**		
Retention time begin	4	0
Retention time end	100	100
Mass range begin	0	0
Mass range end	2000	5000
MS2 mass range begin	0	0
MS2 mass range end	2000	0
**Centroid parameters**		
MS1 tolerance	0.01	0.01
MS2 tolerance	0.025	0.025
**Isotope recognition**		
Maximum charged number	2	2
**Data processing**		
Number of threads	5	5
**Peak detection parameters**		
Smoothing method	LinearWeightedMovingAverage	LinearWeightedMovingAverage
Smoothing level	3	3
Minimum peak width	5	5
Minimum peak height	3000	3000
**Peak spotting parameters**		
Mass slice width	0.1	0.1
Exclusion mass list (mass & tolerance)		
**Deconvolution parameters**		
Sigma window value	0.5	0.5
MS2Dec amplitude cut off	0	0
Exclude after precursor	TRUE	TRUE
Keep isotope until	0.5	0.5
Keep original precursor isotopes	FALSE	FALSE
**MSP file and MS/MS identification setting**		

MSP file	MergedPCDL_AllSpectra_Positive.msp	MergedPCDL_AllSpectra_Positive.msp
Retention time tolerance	100	100
Accurate mass tolerance (MS1)	0.01	0.01
Accurate mass tolerance (MS2)	0.05	0.05
Identification score cut off	80	80
Using retention time for scoring	FALSE	TRUE
Using retention time for filtering	FALSE	FALSE
**Text file and post identification (retention time and accurate mass based) setting**		
Text file		
Retention time tolerance	0.1	0.1
Accurate mass tolerance	0.01	0.01
Identification score cut off	85	85
**Advanced setting for identification**		
Relative abundance cut off	0	0
Top candidate report	FALSE	TRUE
**Adduct ion setting**		
[M+H]+		
[M+NH4]+		
[M+Na]+		
**Alignment parameters setting**		
Reference file	170717_QC1.abf	160522_10_pos_Pest-STD-100.abf
Retention time tolerance	0.05	0.05
MS1 tolerance	0.015	0.015
Retention time factor	0.5	0.5
MS1 factor	0.5	0.5
Peak count filter	0	0
N% detected in at least one group	0	0
Remove feature based on peak height fold-change	FALSE	FALSE
Sample max / blank average	5	5
Sample average / blank average	5	5
Keep identified and annotated metabolites	TRUE	TRUE

Keep removable features and assign the tag for checking	TRUE	TRUE
Gap filling by compulsion	FALSE	TRUE
**Tracking of isotope labels**		
Tracking of isotopic labels	FALSE	FALSE
**Ion mobility**		
Ion mobility data	FALSE	FALSE

Table S2: Labelled internal standards used for retention time alignment in MS-DIAL

Compound	Rt (min)	Rt tol. (min)	m/z	m/z tol. (Da)	Min. Height	Use
Methomyl-D3	6.65	0.2	166.0721	0.025	5000	T
Simazine-D5	9.5	0.2	207.1163	0.025	5000	T
Dimethoate-D6	8.16	0.2	236.0443	0.025	5000	T
Diuron-D6	10.89	0.2	239.0618	0.025	5000	T
Imidacloprid-D4	8.01	0.2	260.0858	0.025	5000	T
Pendimeth-D5	15.9	0.2	287.1775	0.025	5000	T
Boscalid-D4	12.69	0.2	347.0651	0.025	5000	T

Table S3: Parameters used for MS-FLO for adduct feature joining

Sodium adduct joining	Ammonium adduct joining
**[0. Global Parameters]**	**[0. Global Parameters]**
row merging delimiter = _	row merging delimiter = _
**[1. Contaminant Ion Removal]**	**[1. Contaminant Ion Removal]**
enabled = False	enabled = False
**[2. Duplicate Removal]**	**[2. Duplicate Removal]**
enabled = True	enabled = True
mz tolerance = 0.01	mz tolerance = 0.01
retention time tolerance = 0.1	retention time tolerance = 0.1
peak height tolerance = 1.0	peak height tolerance = 1.0
minimum peak match ratio = 0.85	minimum peak match ratio = 0.85
**[3. Isotope Detection]**	**[3. Isotope Detection]**
enabled = True	enabled = True
mz tolerance = 0.01	mz tolerance = 0.01
retention time tolerance = 0.02	retention time tolerance = 0.02
minimum r^2 to match = 0.0	minimum r^2 to match = 0.0
mass shift = 1.003355	mass shift = 1.003355
**[4. Adduct Joiner]**	**[4. Adduct Joiner]**
enabled = True	enabled = True

mz tolerance = 0.01	mz tolerance = 0.01
retention time tolerance = 0.02	retention time tolerance = 0.02
adduct = ['M+H', 'M+Na', 21.98187, 0.0, 0.8]	adduct = ['M+H', 'M+NH4', 17.026547, 0.0, 0.8]

Supplementary Results:

Table S4: Target compound detection in standards and spiked wastewater after MS-DIAL alignment

Compound	Detection Frequency in 100 ppb Standards[1] (%)		Detection Frequency in 20 ppb Spikes[2] (%)		Detection Code[3]	
	MB (n = 4)	SB (n = 7)	MB (n = 10)	SB (n = 12)	MB	SB
Azoxystrobin	100	100	90	100	C	C
Boscalid	100	100	90	100	U	C
Chlorantraniliprole	100	0	80	0	C	
Clomazone	100	100	90	92	C	I
Cyprodinil	100[4]	100	100[4]	100	U	C
DEET	100	100	90	100	C	I
Difenoconazole	100	100	90	100	C	C
Dimethoate	100	100	90	58	U	C
Diuron	100	86	90	50	U	C
Hexazinone	100	100	90	92	C	C
Imidacloprid	100	100	90	83	U	C
Methomyl	100	0	60	8	U	C
Methoxyfenozide	75	100	60	25	U	C
Metolachlor	100	100	90	100	C	C
Pendimethalin	100	100	40	33	U	C
Propanil	100	100	90	75	C	C
Propoxur	100	100	80	67	C	C
Pyriproxyfen	100	100	80	100	C	C
Simazine	100	100	90	67	C	I
Thiacloprid	100	100	90	92	U	C
Thiamethoxam	100	100	80	83	U	C
Thiobencarb	100	100	70	58	I	C
Triclocarban	100	100	40	67	U	C

Triclosan concentration: 10 ppb.
[2] Fipronil concentration: 4 ppb. Pyriproxyfen concentration: 24 ppb. Triclosan concentration: 2 ppb.
[3] C: Compounds Identified correctly; I: Compounds identified as isomers; U: Compounds identified as Unknown but the correct compounds are in the top five hits under Compound Search.

Table S5: Results of feature filtering rules

Filter	Features in SB	Features in MB-unC/C
No filter	63,259	136,938
S/N Ratio	58,007	92,056
Blank	52,399	88,871
Retention time	51,934	85,697
Adduct joining	43,139	82,171
Detections in WW samples[a]	31,989	70,637
Split feature joining	29,772	66,790
Detection frequency at 60% cut-off	3,108	25,822

[a] Many samples were included in the alignment for the purpose of alignment quality control, such as blanks, standards, and spiked wastewater. As a result, some features were present only in these samples and not in the 56-sample subset that was ultimately considered.

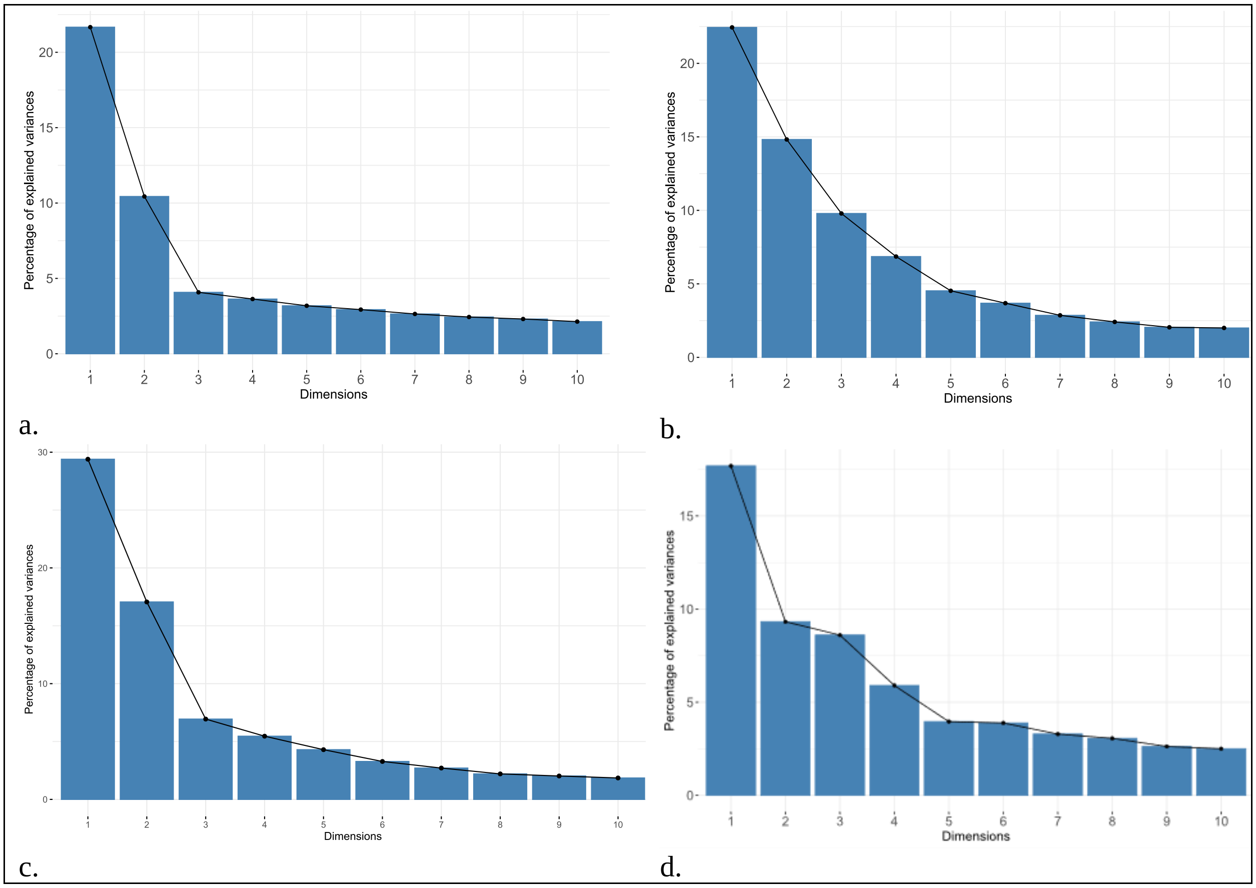

Fig. S2 PCA scree plots for a) single batch (SB), b) uncorrected multi-batch (MB-unC), c) median-ISTD scaled peak height (MB-IS), and d) ComBat-corrected multi-batch (MB-C) show good representation by first three principal components, although suggest that PC's 4 and 5 might be worth investigating as well

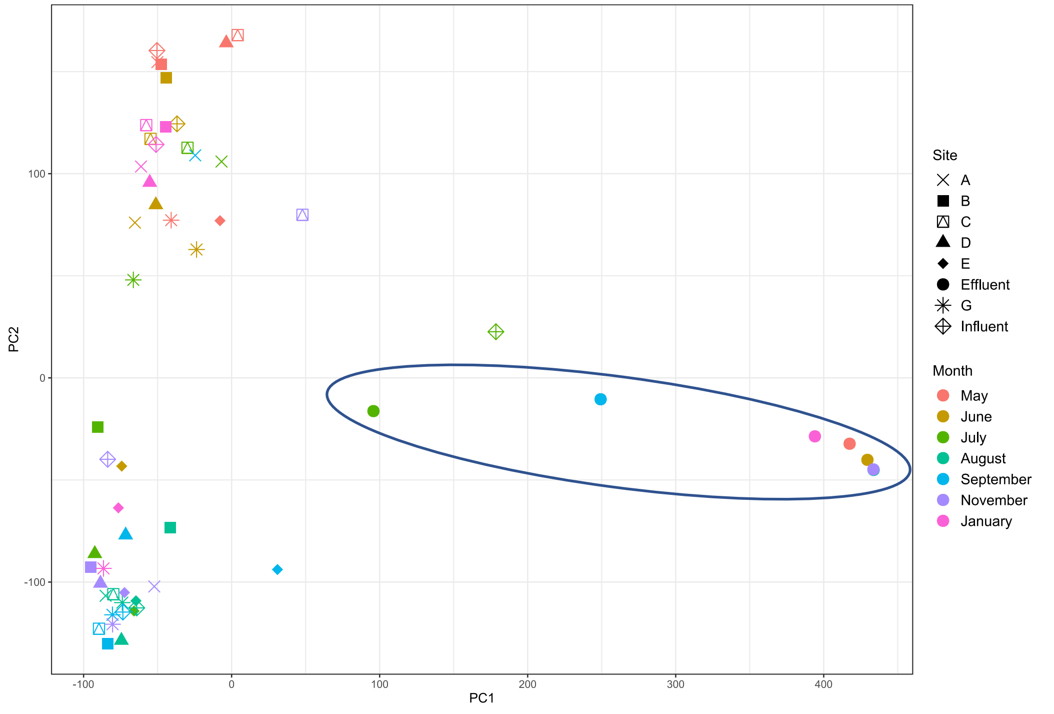

a.

Fig. S3 For SB, a) PCA showed little separation based off sampling month though effluent samples did form their own cluster in PC1 (denoted by oval) from raw WW samples, and b) HCA also shows that effluent samples are very different from influent and trunkline samples

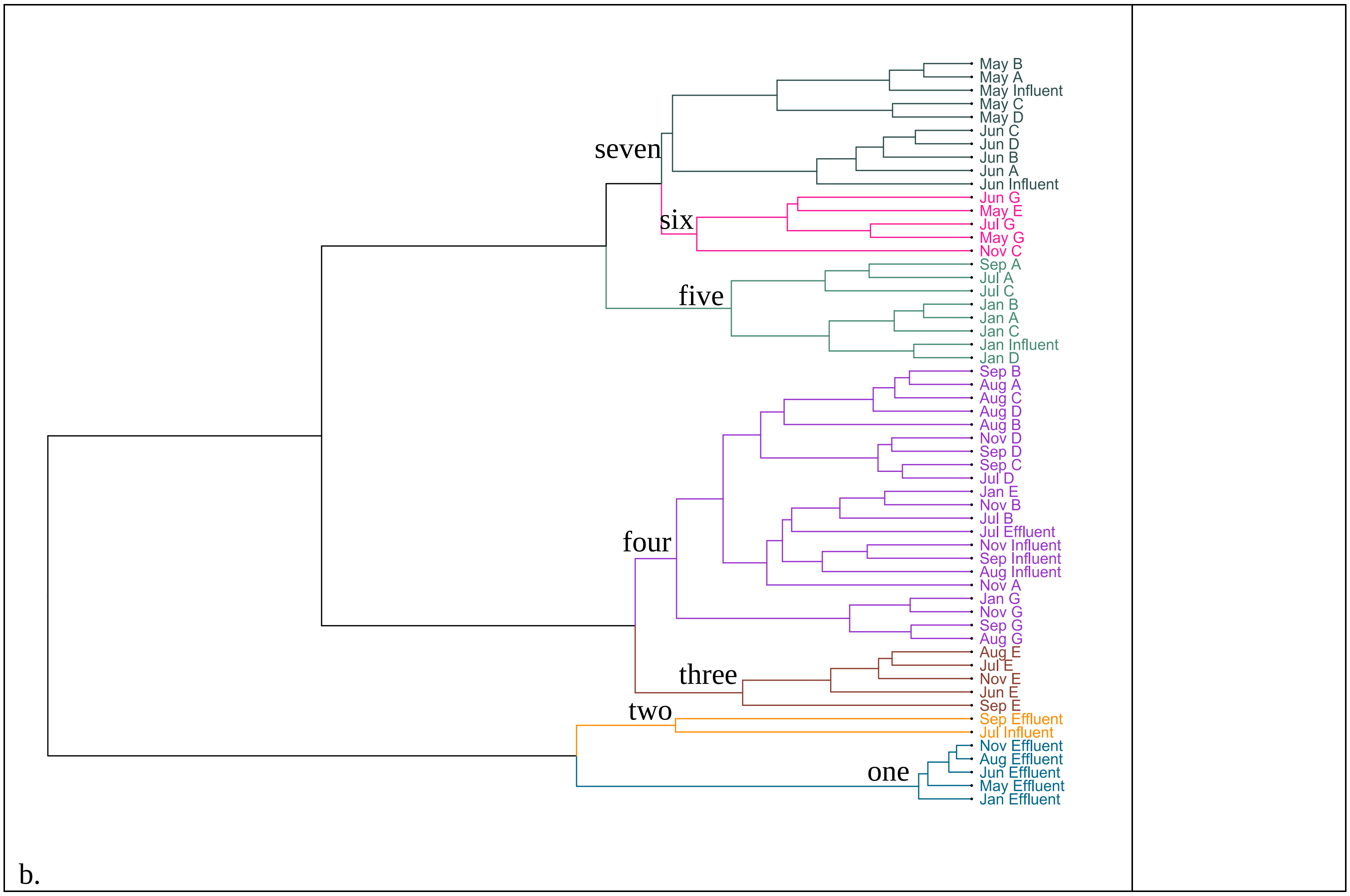

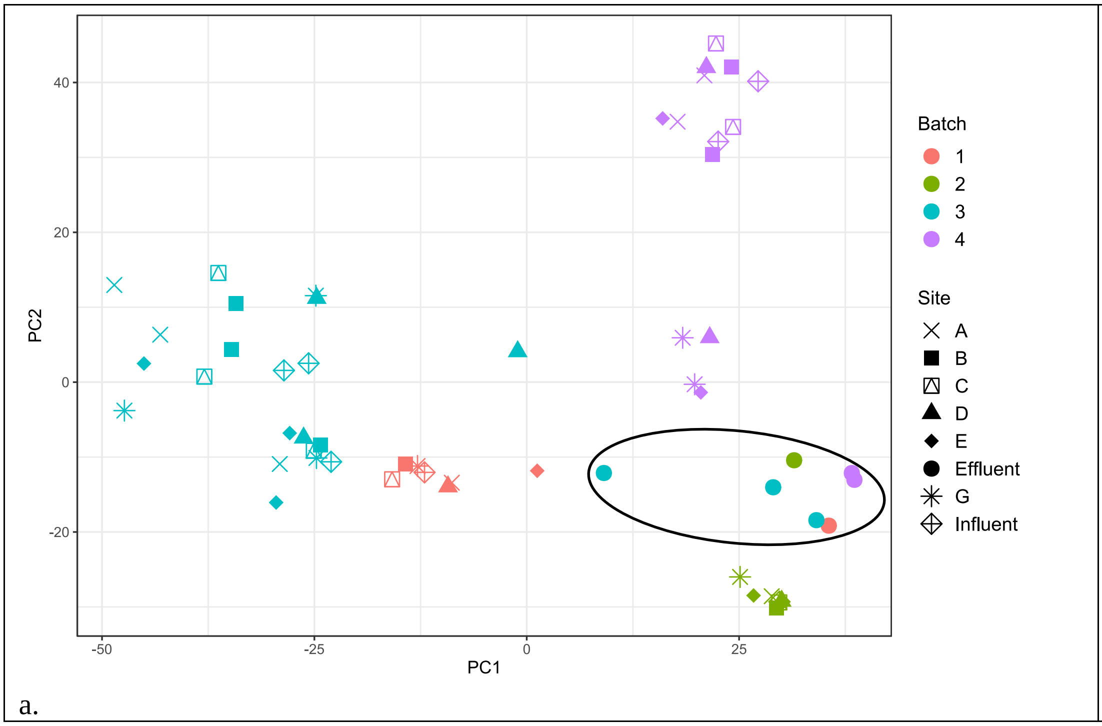

Fig. S4 For MB-IS, a) PCA showed the grouping of effluent samples (circled), which were not entirely distinct in either PC1 or PC2, while raw-WW sample groupings by batch are conserved, and b) HCA shows almost exclusive clustering by batch (denoted by colored bar to right), except for treated effluent samples and cluster six

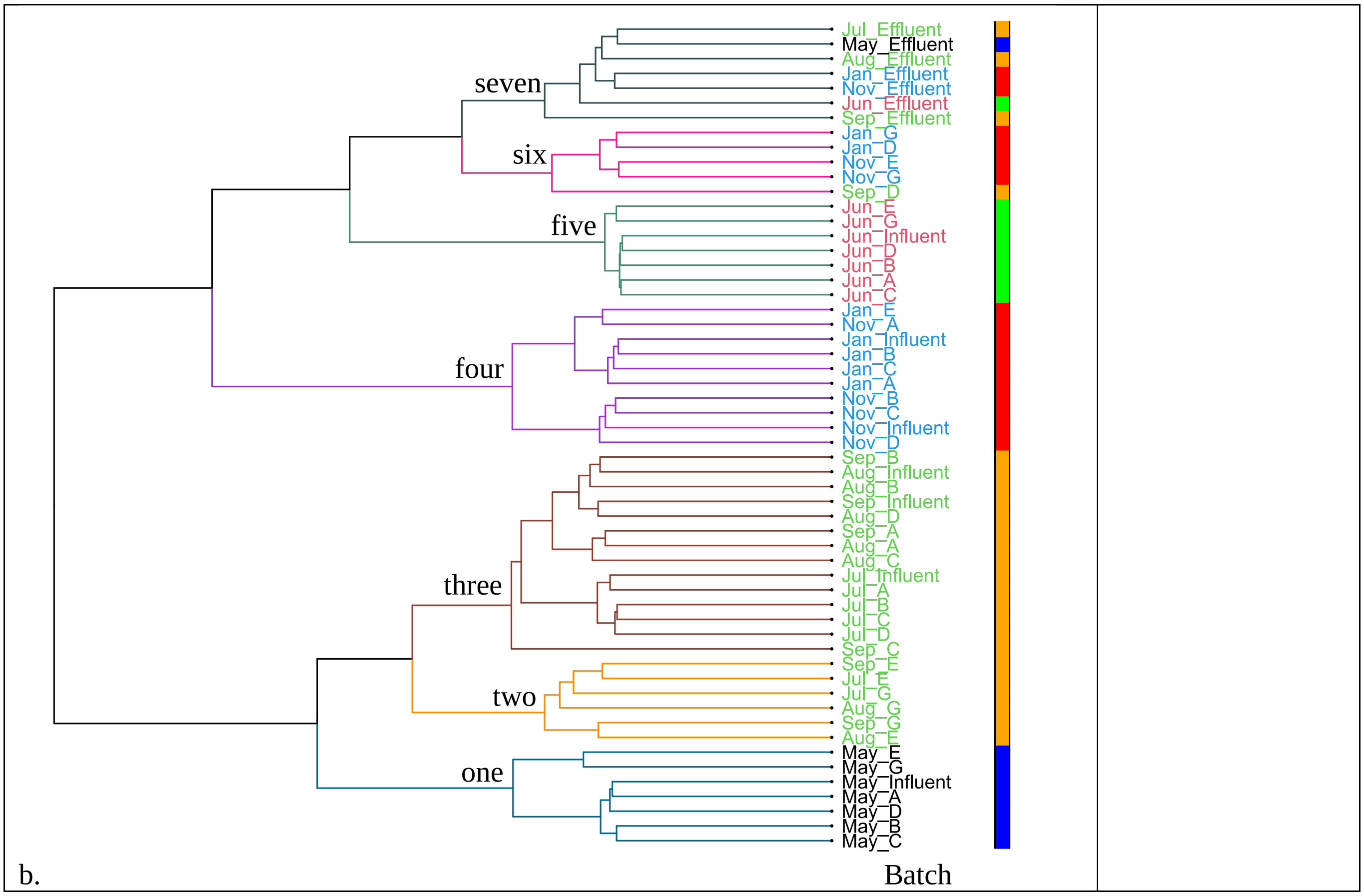
seven
six
five
four
three
two
one
Jul_Effluent
May_Effluent
Aug_Effluent
Jan_Effluent
Nov_Effluent
Jun_Effluent
Sep_Effluent
Jan_G
Jan_D
Nov_E
Nov_G
Sep_D
Jun_E
Jun_G
Jun_Influent
Jun_D
Jun_B
Jun_A
Jun_C
Jan_E
Nov_A
Jan_Influent
Jan_B
Jan_C
Jan_A
Nov_B
Nov_C
Nov_Influent
Nov_D
Sep_B
Aug_Influent
Aug_B
Sep_Influent
Aug_D
Sep_A
Aug_A
Aug_C
Jul_Influent
Jul_A
Jul_B
Jul_C
Jul_D
Sep_C
Sep_E
Jul_E
Jul_G
Aug_G
Sep_G
Aug_E
May_E
May_G
May_Influent
May_A
May_D
May_B
May_C
b.
Batch

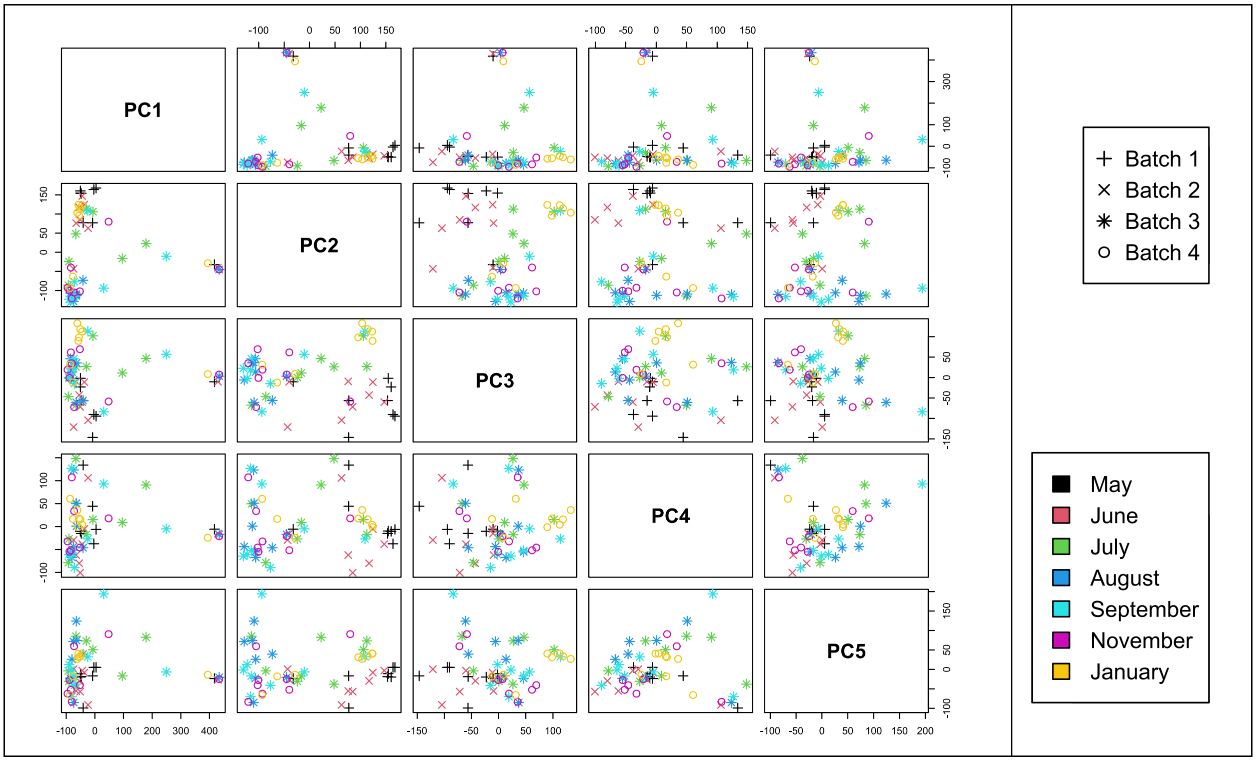
PC1
PC2
PC3
PC4
PC5
Batch 1
Batch 2
Batch 3
Batch 4
May
June
July
August
September
November
January

a.

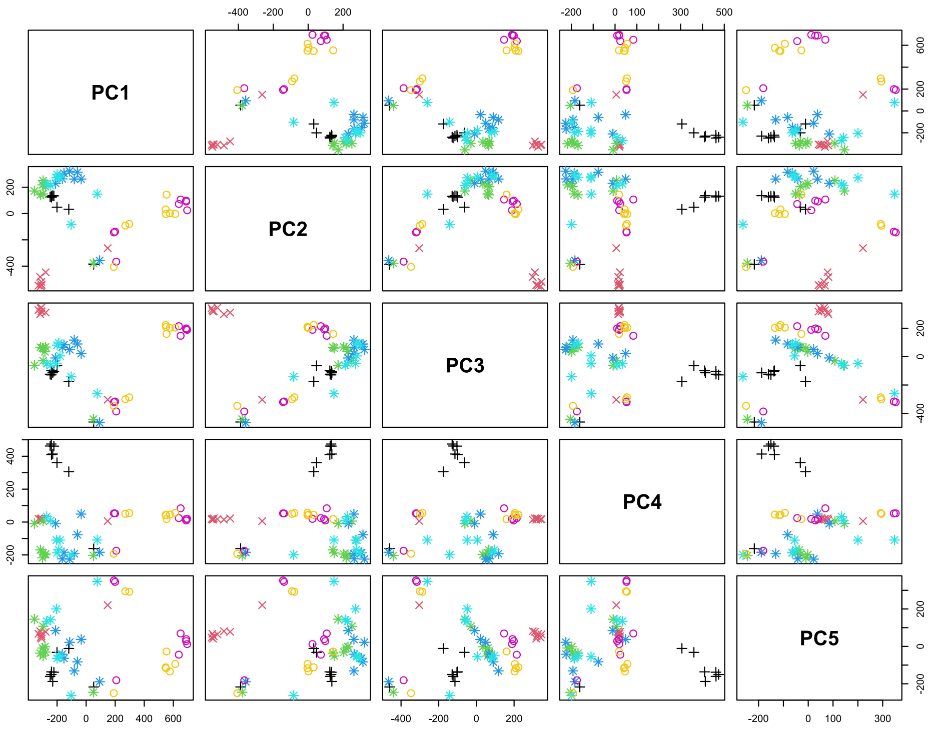
PC1
PC2
PC3
PC4
PC5

b.

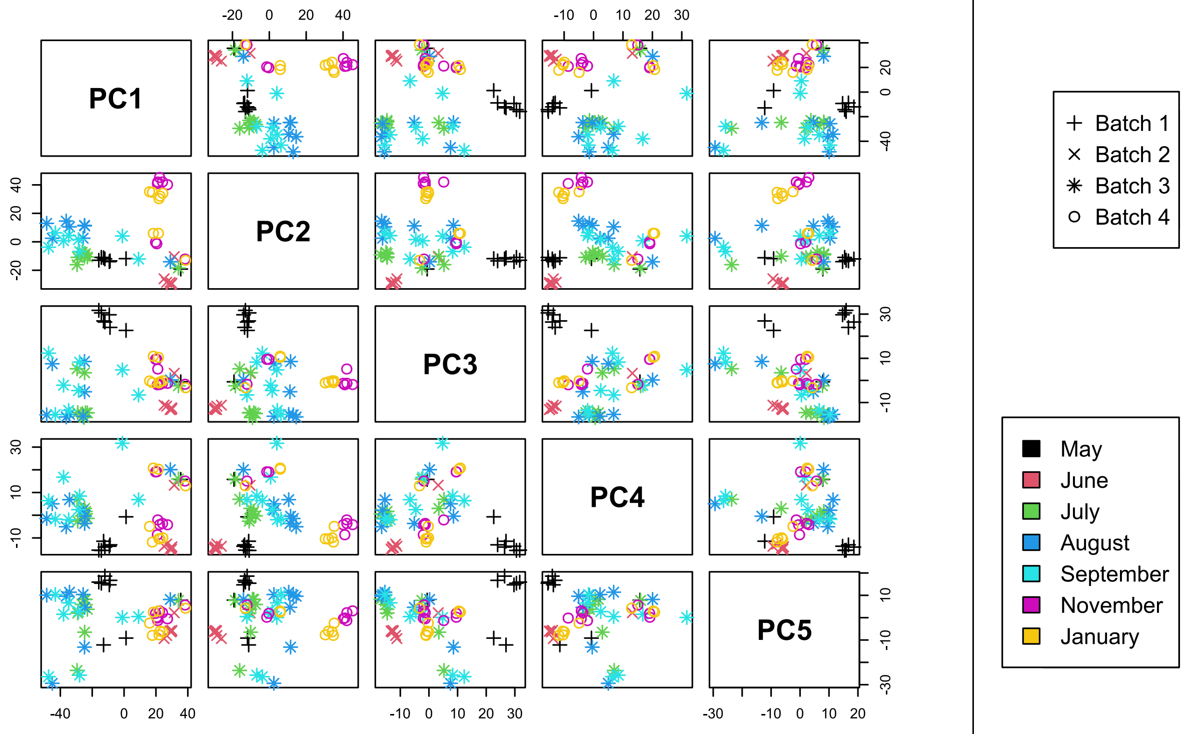
PC1
PC2
PC3
PC4
PC5
Batch 1
Batch 2
Batch 3
Batch 4
May
June
July
August
September
November
January

c.

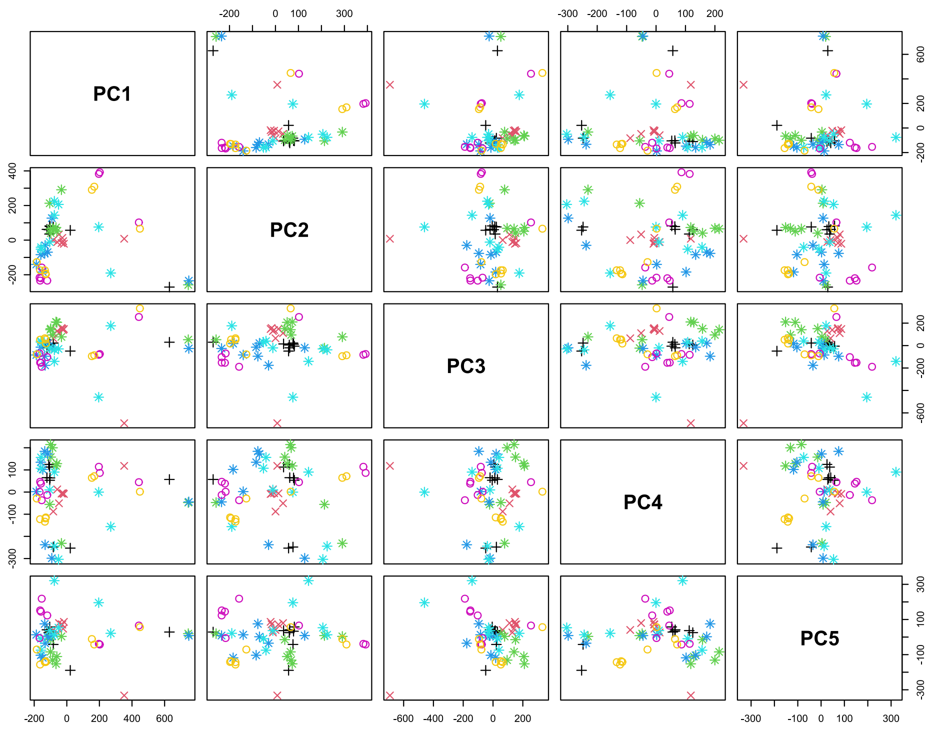

d.

Fig. S5 Matrix pair plots for principal components 1-5 for a) SB, b) MB-unC, c) MB-IS, and d) MB-C

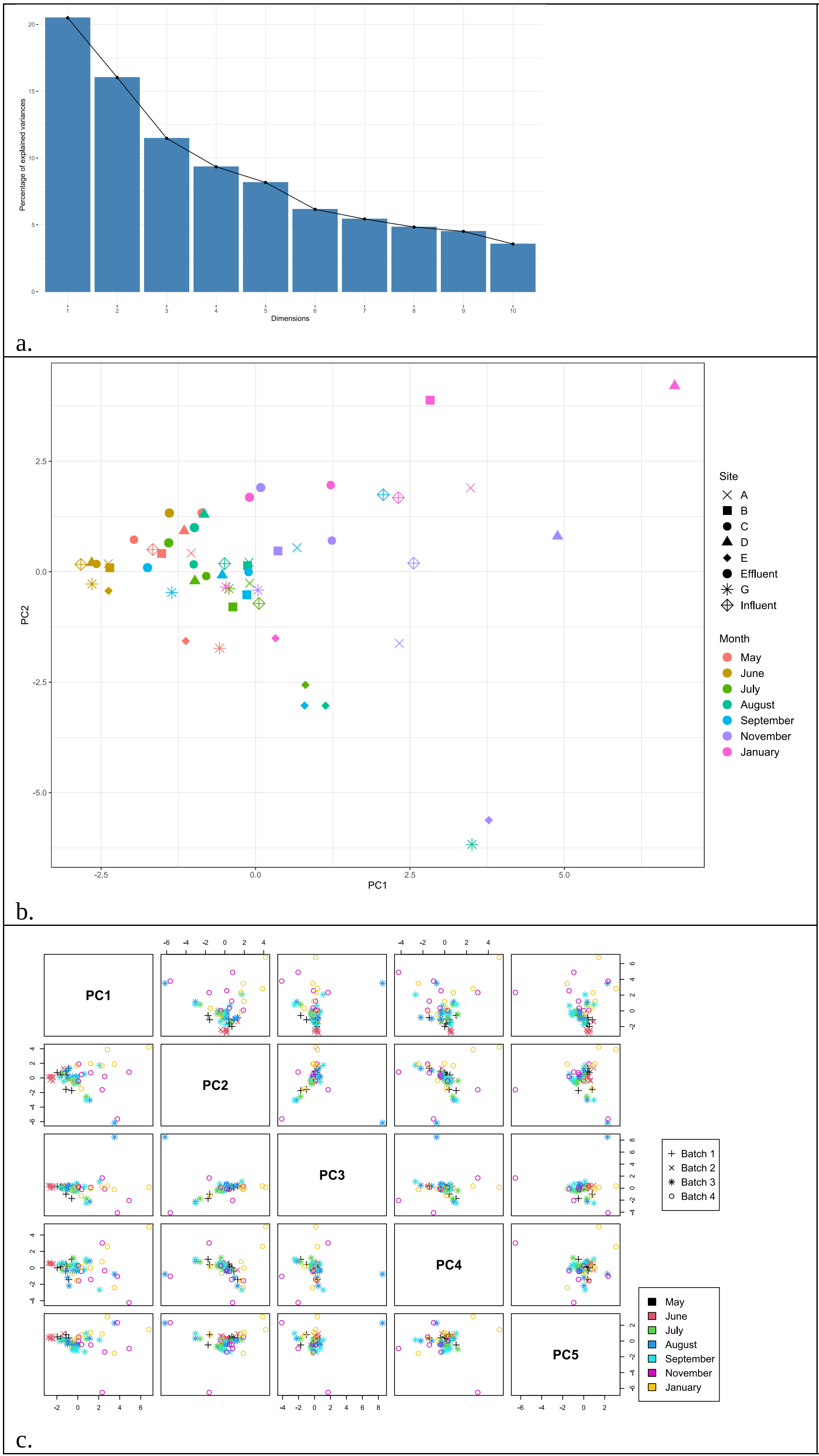

Fig. S6 Using only quantified target pesticides, a) showed approximately a similar explanation of variance as using nontarget features, b) plotting the first two PC's of wastewater samples primarily showed the separation of November and January samples from the rest, c) little separation in PC's 4 and 5

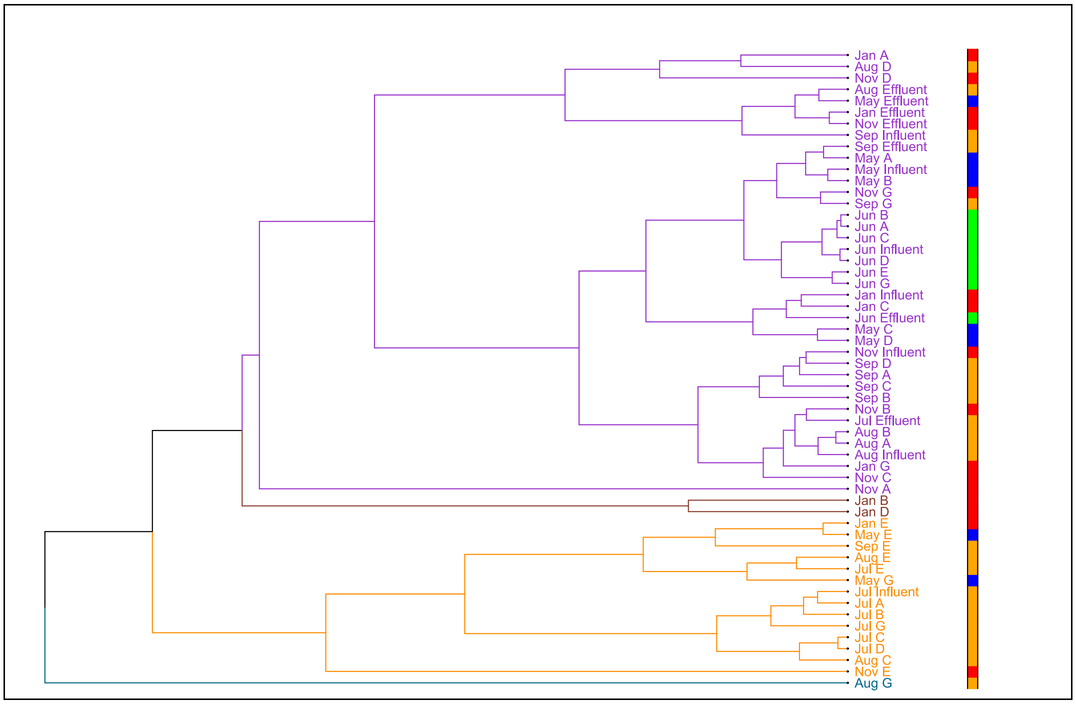

Fig. S7 Conducting HCA with the first 5 principal components indicates (from left to right) one large cluster including trunkline, influent and effluent; a cluster containing a single August trunkline sample (the only detection of chlorothalonil); a cluster of six January and four November samples; and a cluster of four EPA 5-1 plus two other trunkline samples

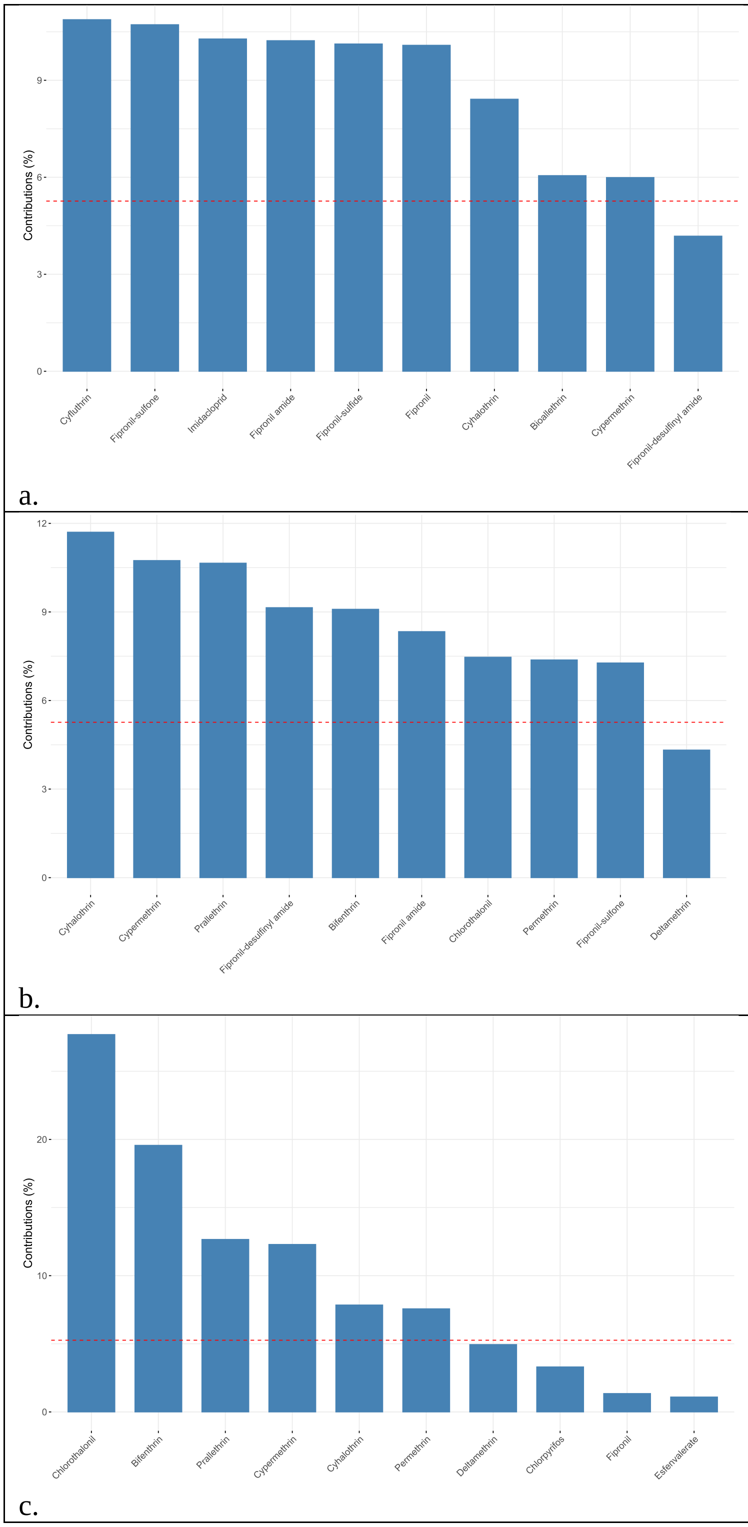

Fig. S8 Top-ten highest variable contributions to principal components for PCA of wastewater set using only target-quantified pesticides in a) the first dimension, b) second dimension, and c) third dimension. The red line denotes the predicted value if variable contribution were uniform (2)

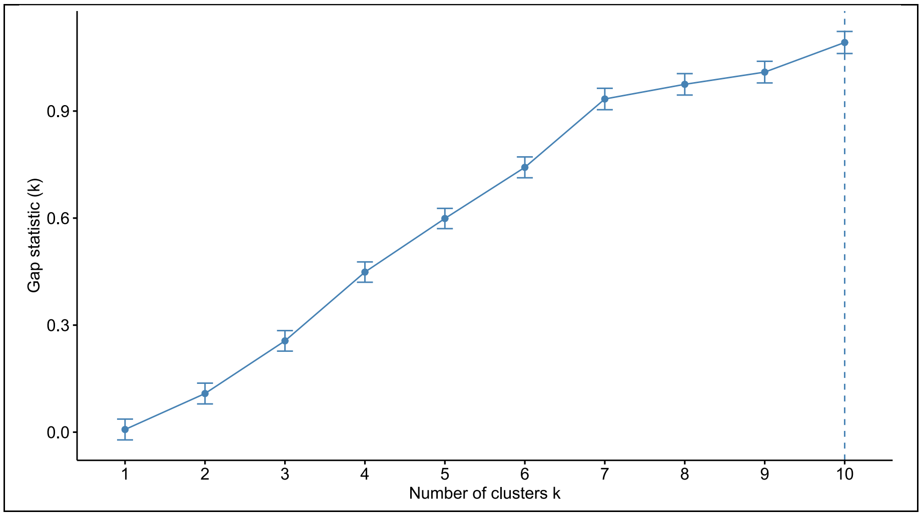

Fig. S9 Gap-statistic plot for MB-unC to determine optimal number of clusters at about 10 clusters

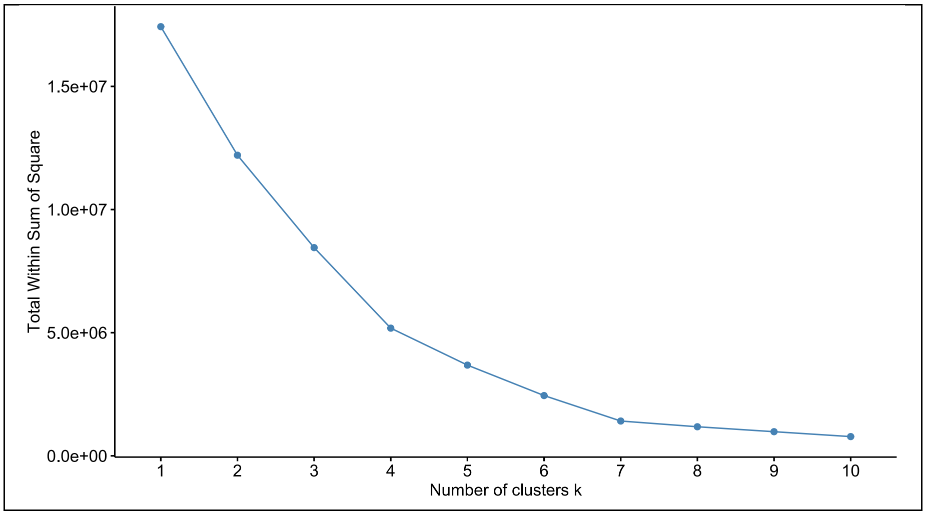

Fig. S10 Total within cluster sum of squares for MB-unC to determine optimal number of clusters, about 7 or 8

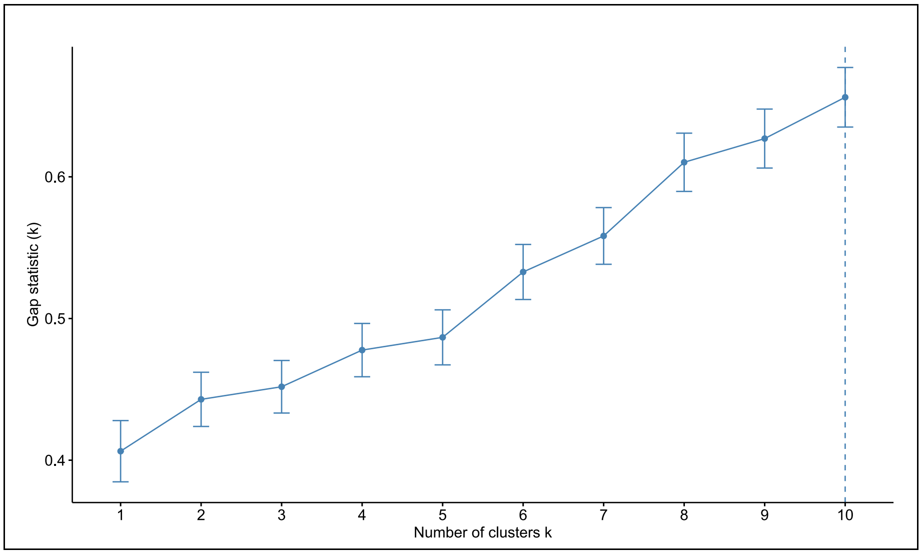

Fig. S11 Gap-statistic plot for MB-C to determine optimal number of clusters at about 10 clusters

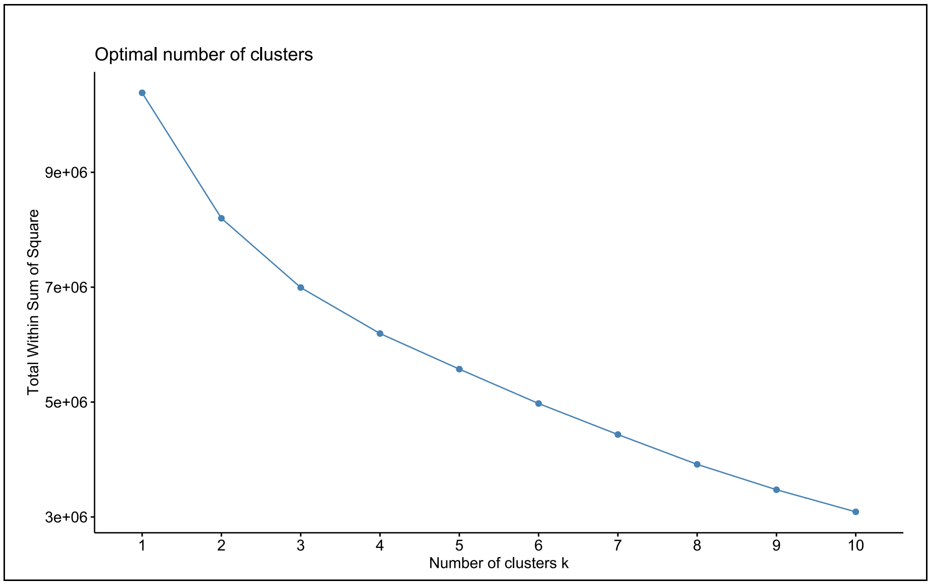

Fig. S12 Total within-sum-squares plot for MB-C to determine optimal number of clusters at about 10 clusters

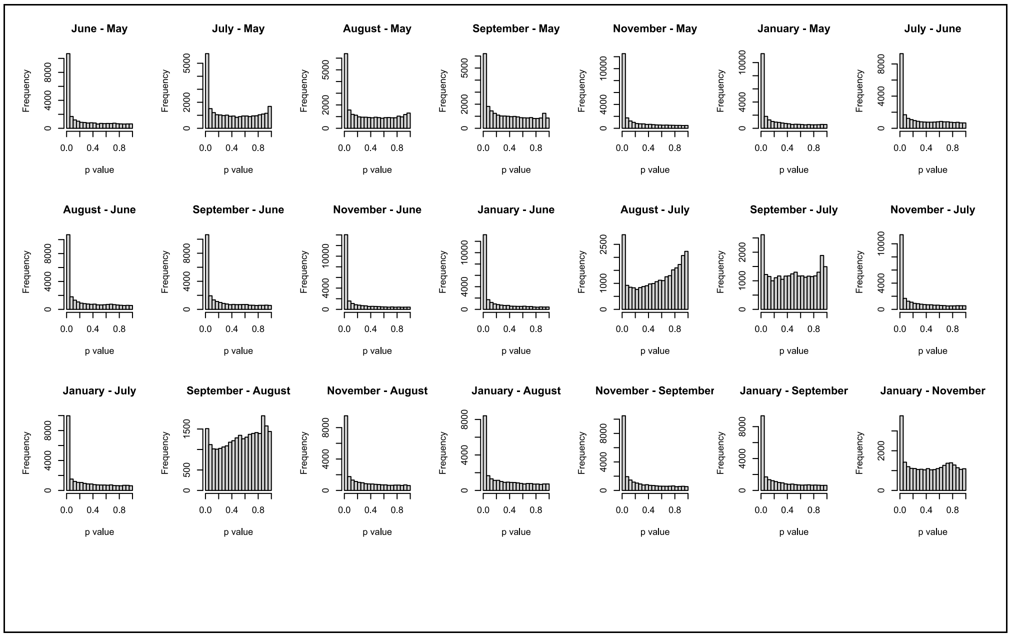

Fig. S13 Histograms of p-values from contrasts by months of MB-unC shows many features are significantly different between months

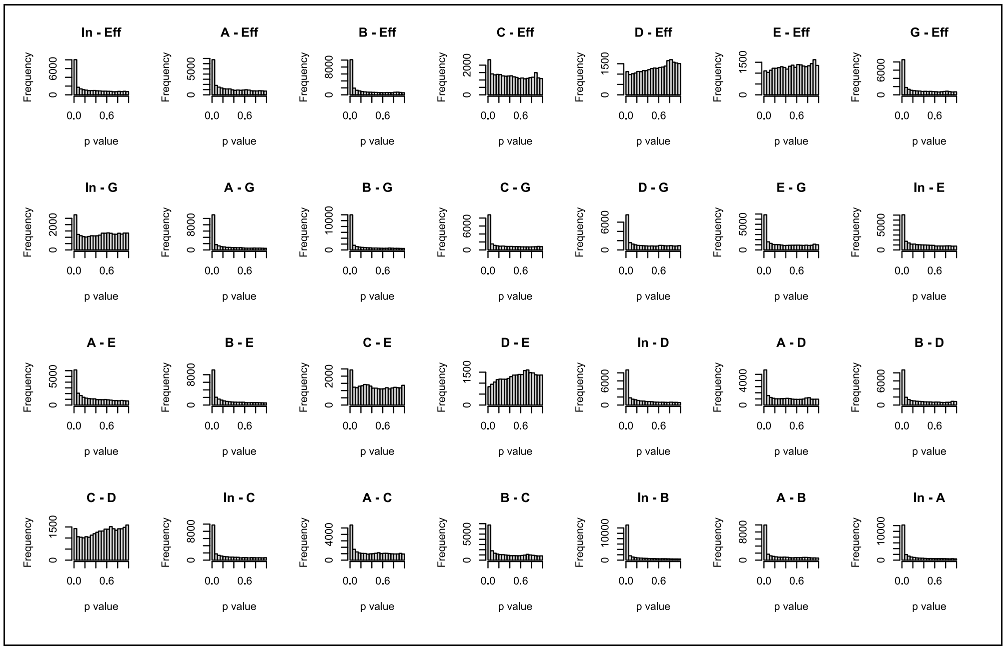

Figure S14. Histograms of p-values from contrasts by sampling site of MB-unC shows significant differences between sampling sites, but also that samples such as C, D, and E are more like effluent, and C – D and D – E are similar

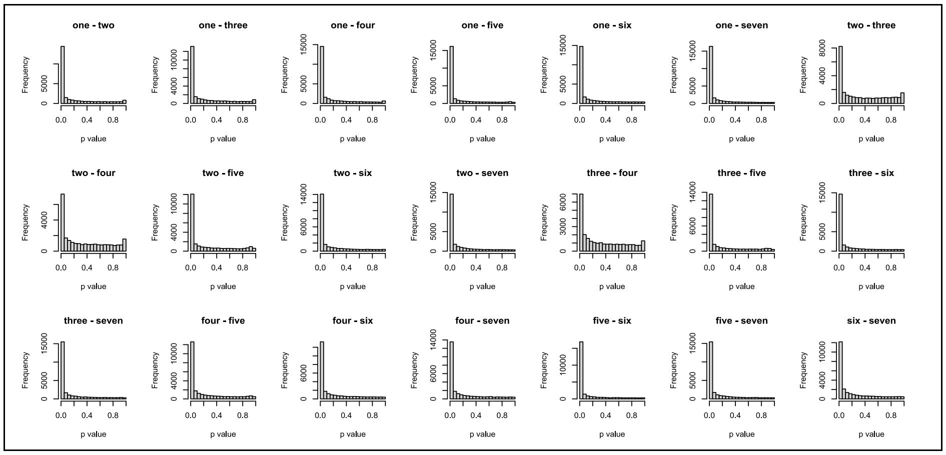

Fig. S15 Histograms of p-values from contrasts by HCA cluster shows high numbers of significantly different features for MB-unC

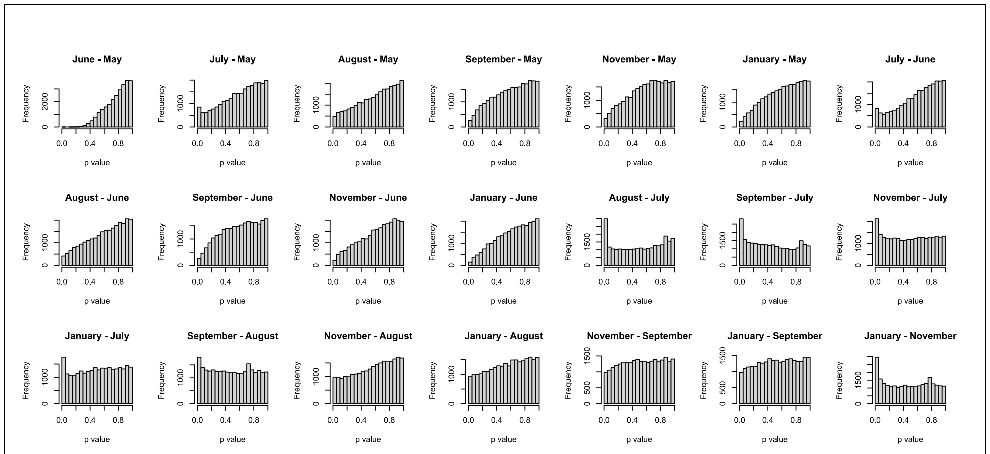

Fig. S16 Histograms of p-values from contrasts by month of MB-C shows only a few months with a large number of significantly different features, unlike for MB-unC (Fig. S13)

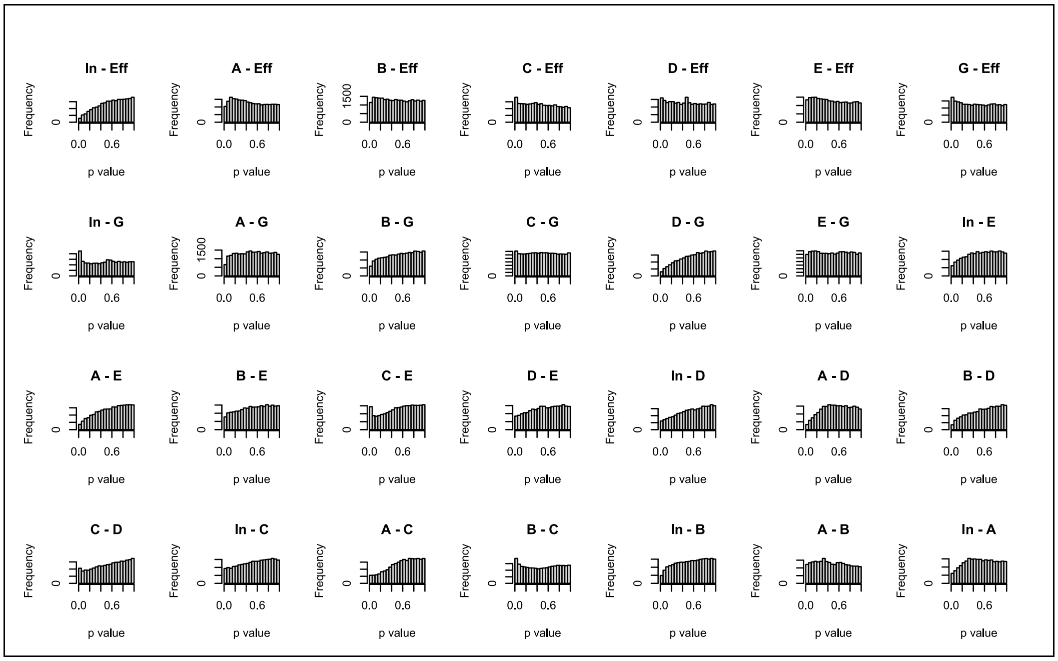

Fig. S17 Histograms of p-values from contrasts by site for MB-C shows very few significantly different features, dissimilar from MB-unC (Fig. S14)

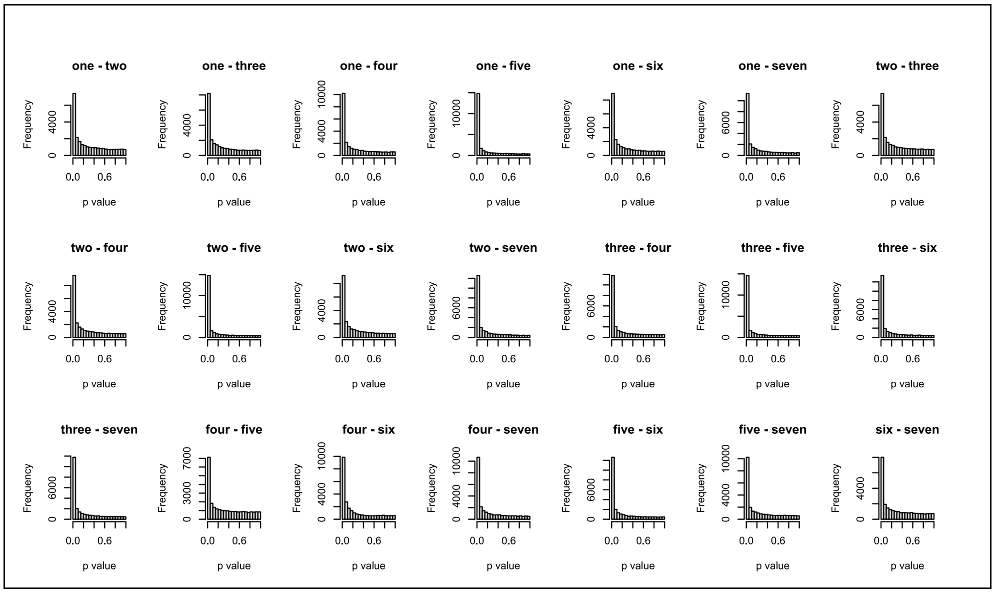

Fig. S18 Histograms of p-values from contrasts by HCA cluster shows high numbers of significantly different features for MB-C

Table S6. Counts of features with $p_{adj} < 0.05$ for MB-unC and MB-C, by HCA cluster, Site, and Month

Contrast	MB-unC	MB-C	Contrast	MB-unC	MB-C	Contrast	MB-unC	MB-C
In - A	1434		January - November	179	8	one - two	1825	555
In - B	1658		January - September	917		one - three	1509	655
In - C	1016		January - August	821		one - four	1699	1084
In - D	837		January - July	1062	3	one - five	2191	2125
In - E	486		January - June	1677		one - six	1949	1002
In - G	42		January - May	1300		one - seven	2220	1396
In - Eff	691		November - September	1012		two - three	848	463
A - B	1155		November - August	931		two - four	613	945
A - C	353		November - July	1286		two - five	1607	2109
A - D	353		November - June	1823		two - six	2031	1126
A - E	403		November - May	1471		two - seven	1941	1571
A - G	1245		September - August	8	4	three - four	492	1400
A - Eff	556		September - July	98	86	three - five	1804	2104
B - C	530		September - June	1275		three - six	2212	1834
B - D	856		September - May	498		three - seven	2143	1460
B - E	948		August - July	154	132	four - five	1620	627
B - Eff	1092		August - June	1276		four - six	1898	975
C - E	61		August - May	519		four - seven	1856	1194
C - G	1714		July - June	1071		five - six	2412	1586
C - Eff	43		July - May	457		five - seven	2166	1190
D - G	643		June - May	1290		six - seven	1513	796
E - G	529							
G - Eff	755							

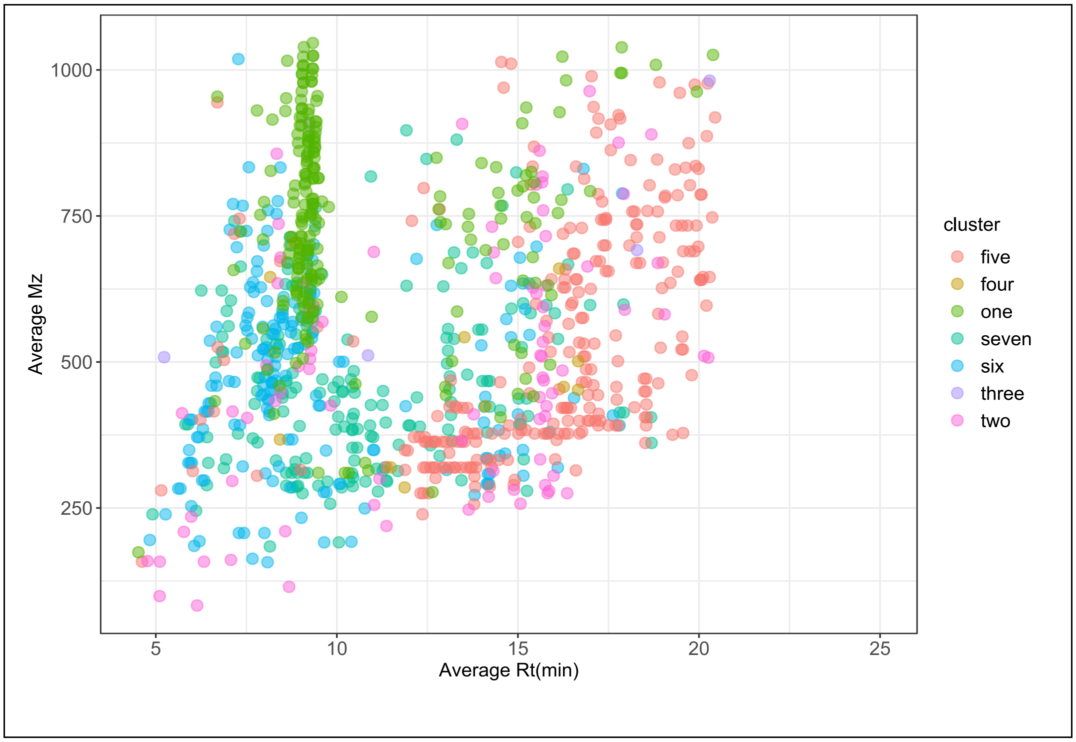

Fig. S19 M/z to RT plot of features found to be significantly lower in a single cluster (denoted by color) in MB-unC dataset

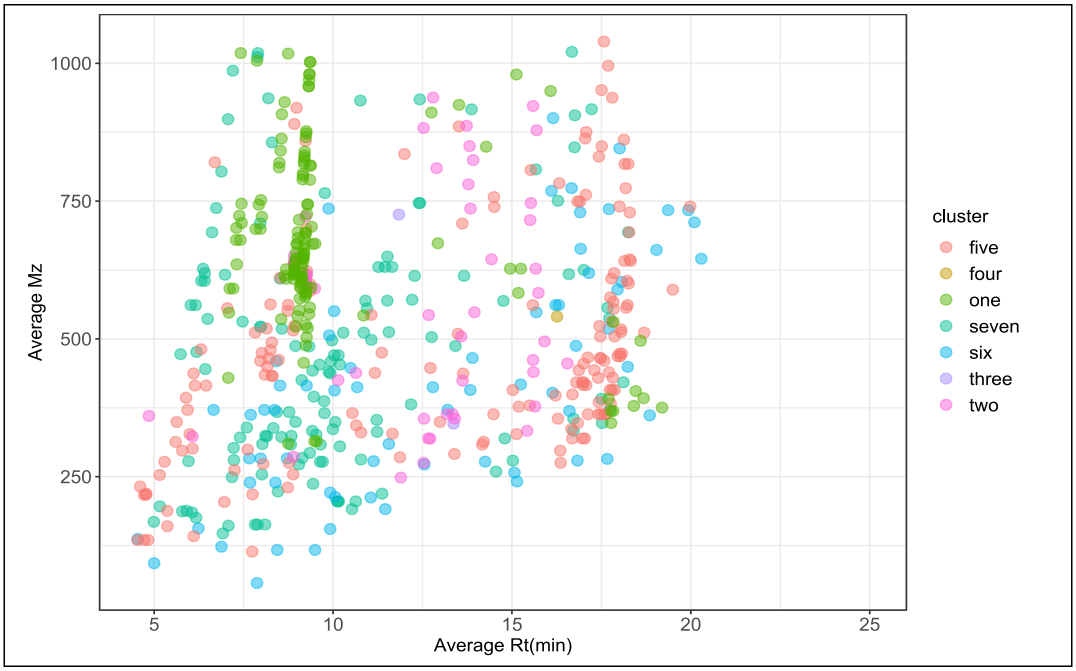

Fig. S20 M/z to RT plot of features found to be significantly higher in a single cluster (denoted by color) in MB-unC dataset

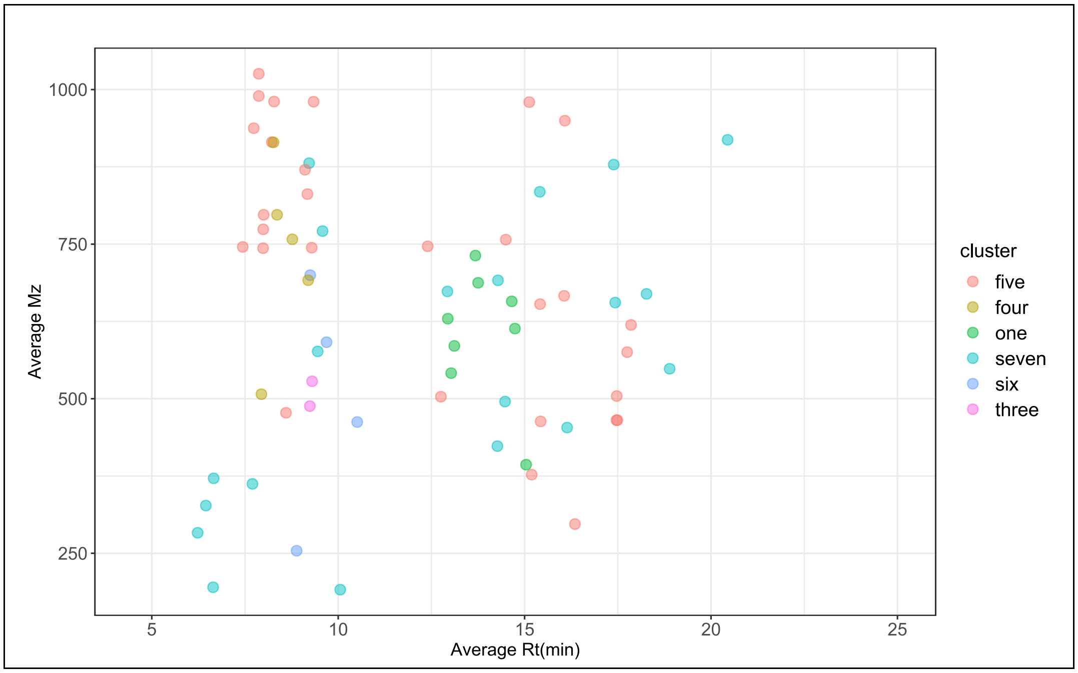

Fig. S21 M/z to RT plot of features found to be significantly higher in a single cluster (denoted by color) in MB-C dataset

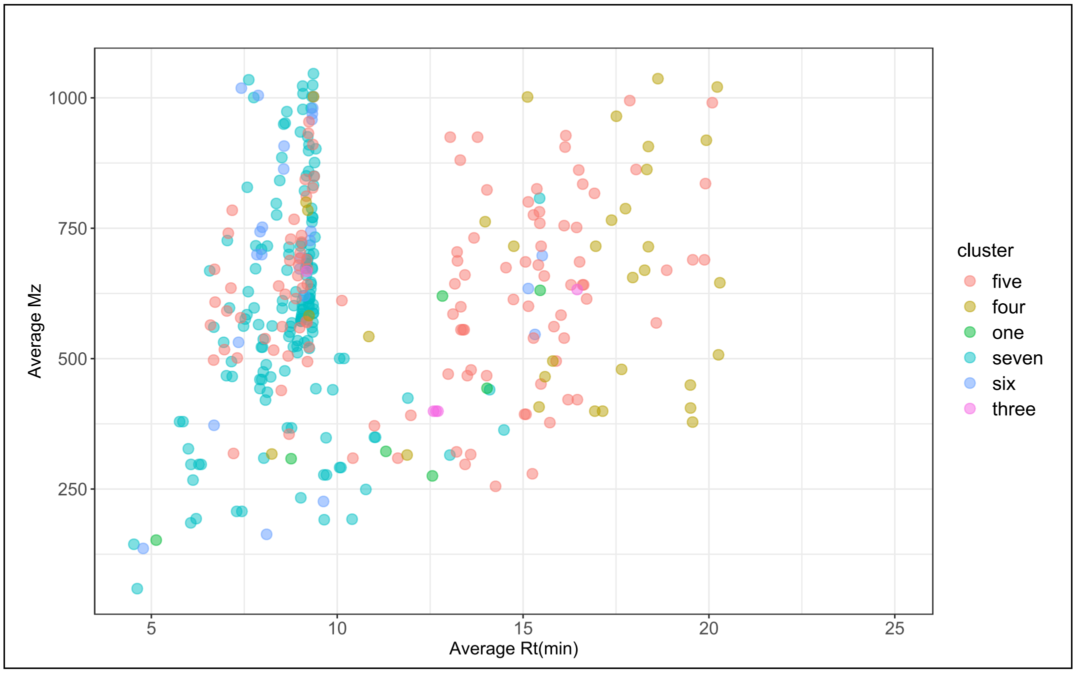

Fig. S22 M/z to RT plot of features found to be significantly lower in a single cluster (denoted by color) in MB-C dataset

References

1. Moschet C, Lew BM, Hasenbein S, Anumol T, Young TM. LC- and GC-QTOF-MS as Complementary Tools for a Comprehensive Micropollutant Analysis in Aquatic Systems. Environ Sci Technol. 2017;51(3):1553–61.

2. Kassambara A, Mundt F. factoextra: Extract and Visualize the Results of Multivariate Data Analyses. R Packag version 107 [Internet]. 2020; Available from: https://cran.r-project.org/package=factoextra
